# Supplementary material for: Allosteric modulators enhance agonist efficacy by increasing the residence time of a GPCR in the active state
Source: Nat Commun. 2021 Sep 14;12:5426. doi: 10.1038/s41467-021-25620-5 (PMC8440590; doi:10.1038/s41467-021-25620-5)
Supplement: Supplementary file 1 — Supplmentary informations [file 41467_2021_25620_MOESM1_ESM.pdf]

Supplementary Information

Allosteric modulators enhance agonist efficacy by increasing the residence time of a GPCR in the active state

Anne-Marinette Cao[1#†,] Robert B. Quast[1#], Fataneh Fatemi[2,1], Philippe Rondard[3,], Jean-Philippe Pin[3,#], Emmanuel Margeat[1,*]

[1] *Centre de Biologie Structurale (CBS), Univ. Montpellier, CNRS, INSERM, Montpellier, France*

[2] *Protein Research Center, Shahid Beheshti University, Tehran, Iran*

[3] *Institut de Génomique Fonctionnelle, Univ. Montpellier, CNRS, INSERM, Montpellier, France*

[#] These authors contributed equally to this work.

* : Corresponding authors: jean-philippe.pin@igf.cnrs.fr, margeat@cbs.cnrs.fr

[†] Present address: École Polytechnique Fédérale de Lausanne (EPFL), SB ISIC LCBM, Station 6, 1015 Lausanne, Switzerland

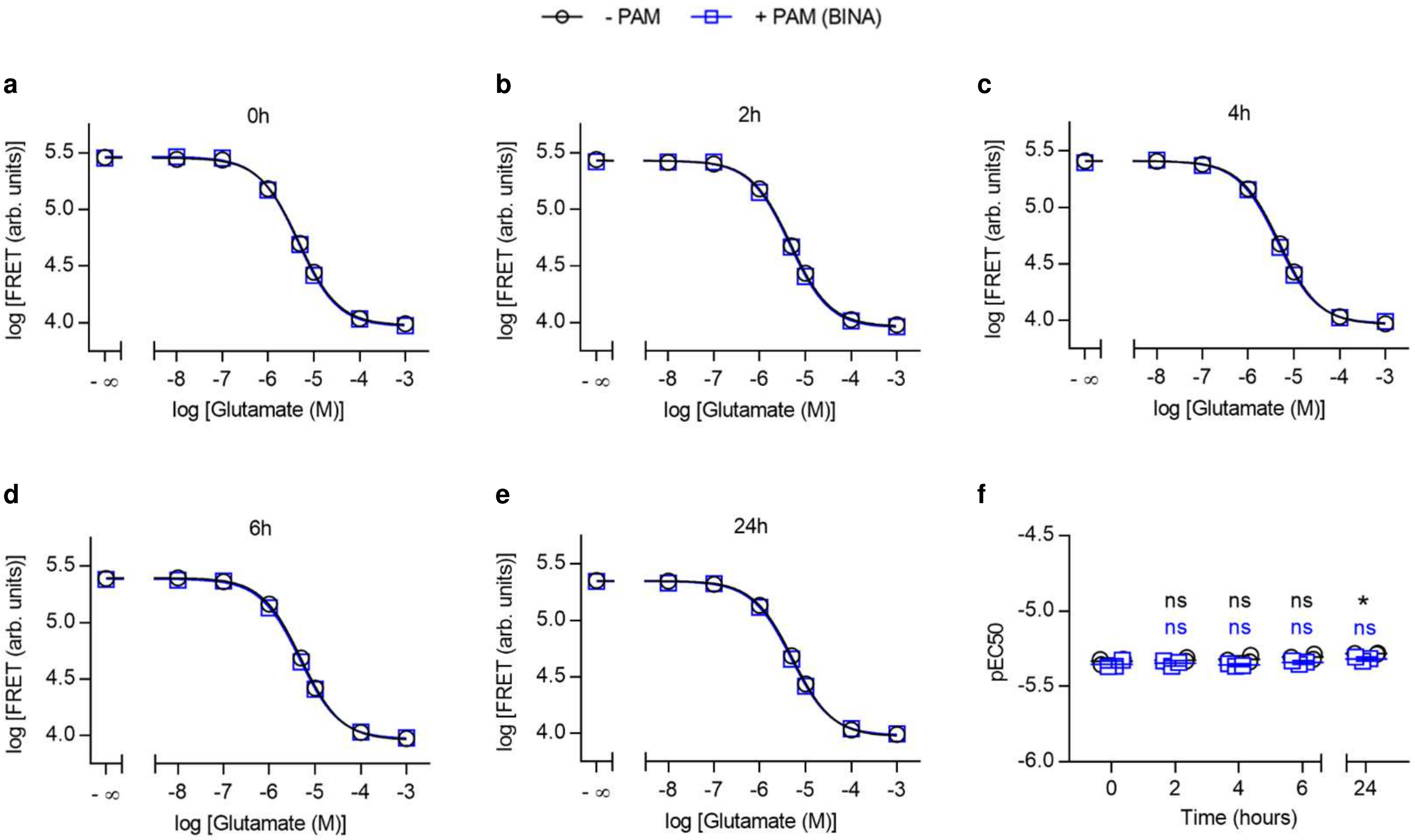

Supplementary Figure 1: Functional assessment of mGlu2 in 0.05% IGEPAL by LRET. a-e) Dose-response curves of Glutamate in the absence (- PAM, black circles) and presence of 10 µM BINA (+ PAM, blue squares) recorded after different time intervals of sample storage at room temperature. Data represent the mean of three biological replicates +/- SD, each measured in triplicates. f) Change of pEC_{50} values over time, obtained from dose-response curves in a-e. Statistical differences of pEC_{50} values for Glu (black) and Glu + BINA (blue) compared to time 0h were determined using two-sided unpaired t-tests and are given as p_{Glu-2h} = 0.65 (ns), p_{Glu-4h} = 0.49 (ns), p_{Glu-6h} = 0.14 (ns), $p_{Glu-24h}$ = 0.014 (*), $p_{Glu+BINA-2h}$ = 0.68 (ns), $p_{Glu+BINA-4h}$ = 0.72 (ns), $p_{Glu+BINA-6h}$ = 0.47 (ns), $p_{Glu+BINA-24h}$ = 0.086 (ns), n = 3 independent biological samples examined over 3 independent experiments. Individual biological replicates are given together with the mean +/- SD.

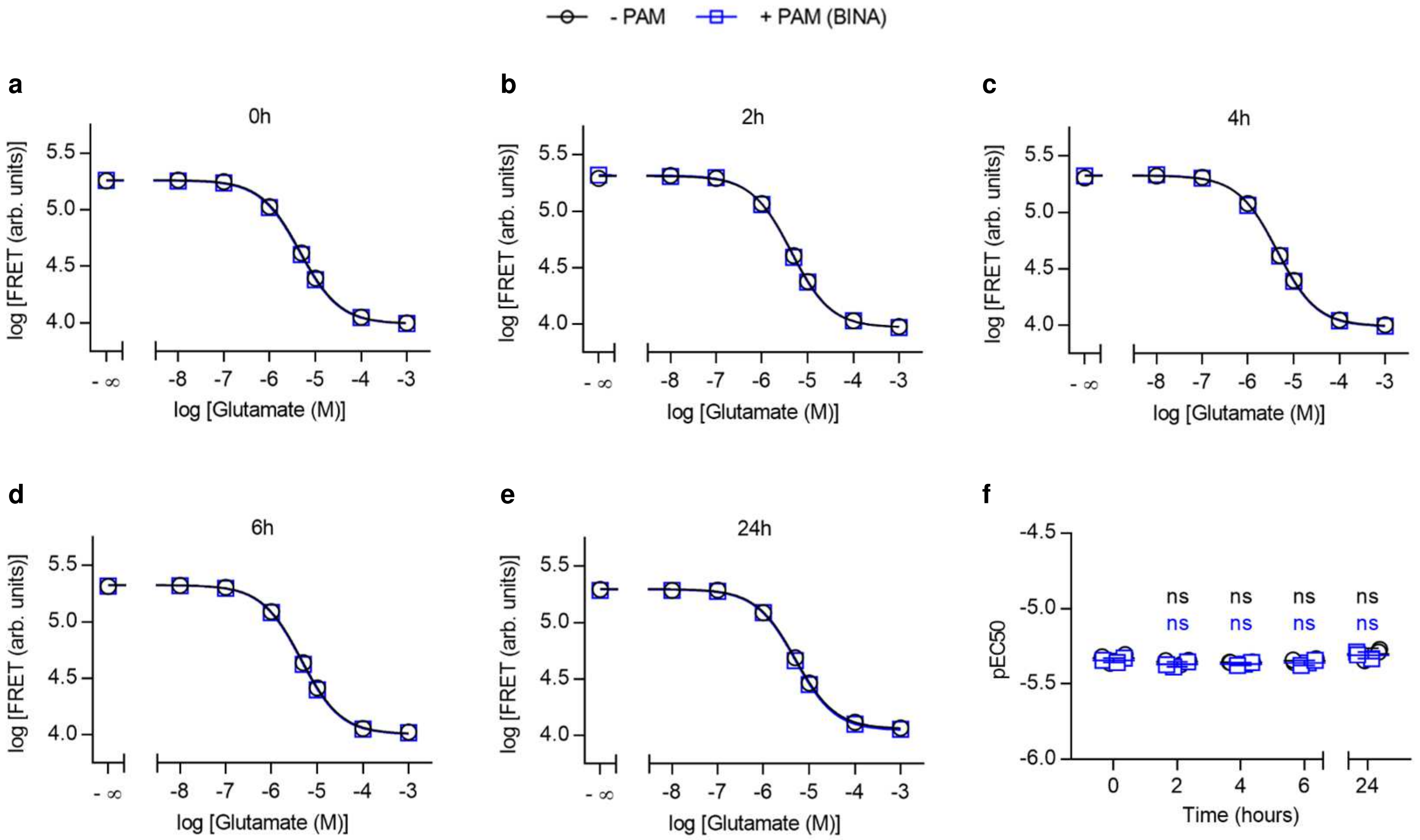

Supplementary Figure 2: Functional assessment of mGlu2 in 0.05% DDM by LRET. a-e) Dose-response curves of Glutamate in the absence (- PAM, black circles) and presence of 10 µM BINA (+ PAM, blue squares) recorded after different time intervals of sample storage at room temperature. Data represent the mean of three biological replicates +/- SD, each measured in triplicates. f) Change of pEC_{50} values over time, obtained from dose-response curves in a-e. Statistical differences of pEC_{50} values for Glu (black) and Glu + BINA (blue) compared to time 0h were determined using two-sided unpaired t-tests and are given as $p_{Glu\text{-}2h}$ = 0.29 (ns), $p_{Glu\text{-}4h}$ = 0.2 (ns), $p_{Glu\text{-}6h}$ = 0.46 (ns), $p_{Glu\text{-}24h}$ = 0.38 (ns), $p_{Glu+BINA\text{-}2h}$ = 0.0995 (ns), $p_{Glu+BINA\text{-}4h}$ = 0.068 (ns), $p_{Glu+BINA\text{-}6h}$ = 0.25 (ns), $p_{Glu+BINA\text{-}24h}$ = 0.086 (ns), n = 3 independent biological samples examined over 3 independent experiments. Individual biological replicates are given together with the mean +/- SD.

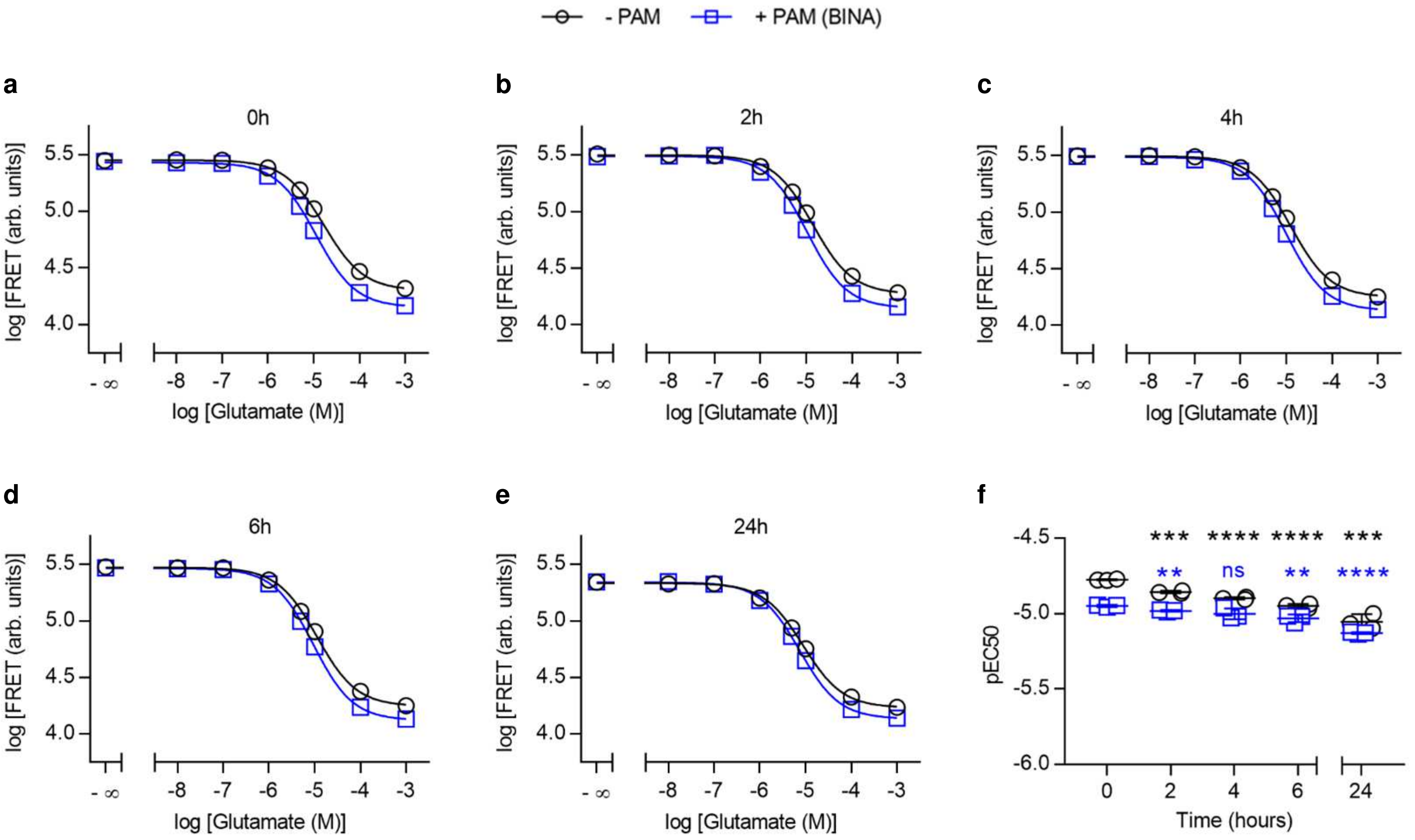

Supplementary Figure 3: Functional assessment of mGlu2 in 0.05% DDM + 0.008% CHS by LRET. a-e) Dose-response curves of Glutamate in the absence (- PAM, black circles) and presence of 10 µM BINA (+ PAM, blue squares) recorded after different time intervals of sample storage at room temperature. Data represent the mean of three biological replicates +/- SD, each measured in triplicates. f) Change of pEC_{50} values over time, obtained from dose-response curves in a-e. Statistical differences of pEC_{50} values for Glu (black) and Glu + BINA (blue) compared to time 0h were determined using two-sided unpaired t-tests and are given as $p_{Glu\text{-}2h}$ = 0.00011 (***), $p_{Glu\text{-}4h}$ = 0.00002 (****), $p_{Glu\text{-}6h}$ = 0.00003 (****), $p_{Glu\text{-}24h}$ = 0.00067 (***), $p_{Glu+BINA\text{-}2h}$ = 0.0044 (**), $p_{Glu+BINA\text{-}4h}$ = 0.066 (ns), $p_{Glu+BINA\text{-}6h}$ = 0.0056 (**), $p_{Glu+BINA\text{-}24h}$ = 0.000006 (****), n = 3 independent biological samples examined over 3 independent experiments. Individual biological replicates are given together with the mean +/- SD.

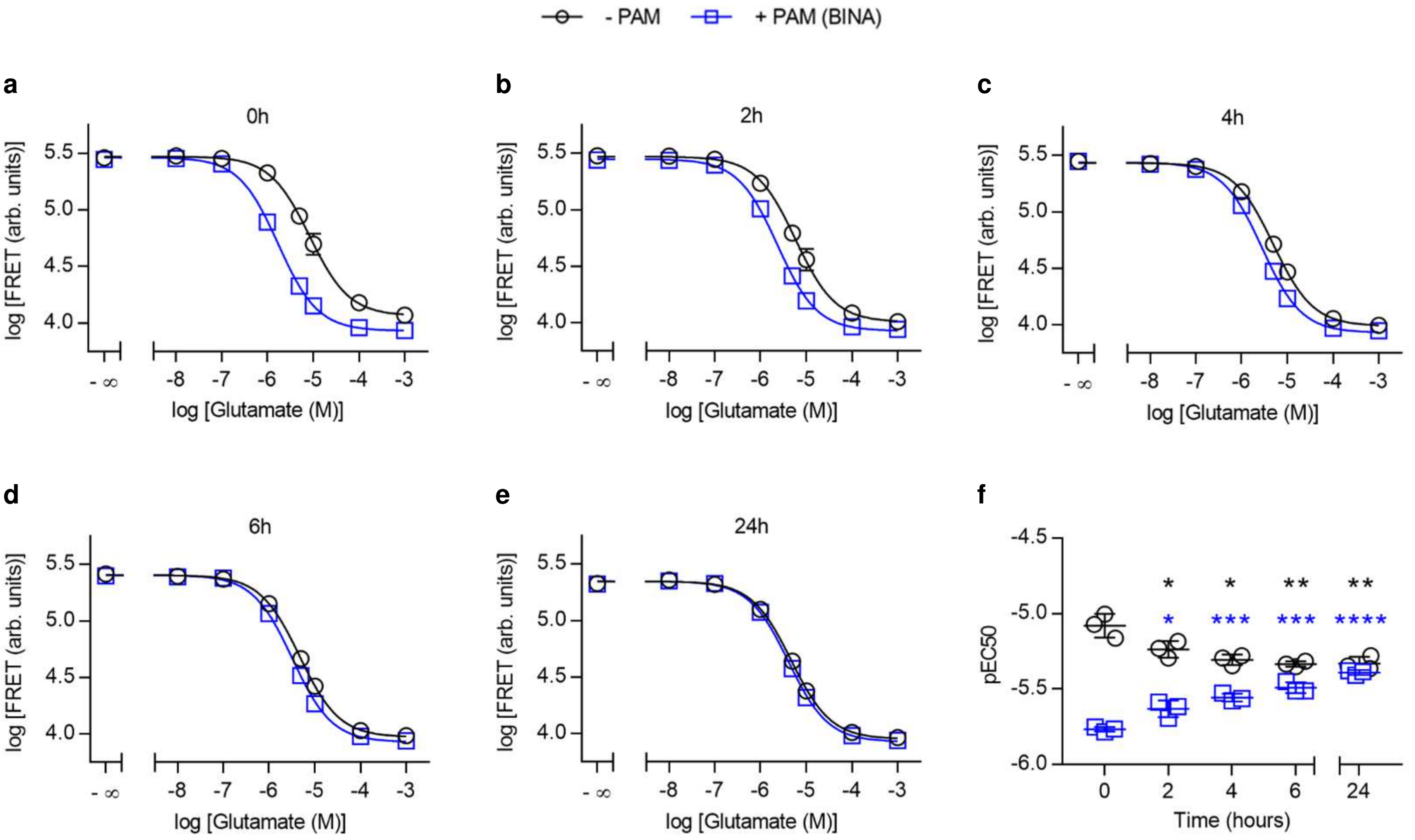

Supplementary Figure 4: Functional assessment of mGlu2 in 0.005% LMNG by LRET. a-e) Dose-response curves of Glutamate in the absence (- PAM, black circles) and presence of 10 µM BINA (+ PAM, blue squares) recorded after different time intervals of sample storage at room temperature. Data represent the mean of three biological replicates +/- SD, each measured in triplicates. f) Change of pEC_{50} values over time, obtained from dose-response curves in a-e. Statistical differences of pEC_{50} values for Glu (black) and Glu + BINA (blue) compared to time 0h were determined using two-sided unpaired t-tests and are given as $p_{Glu\text{-}2h}$ = 0.048 (*), $p_{Glu\text{-}4h}$ = 0.011 (*), $p_{Glu\text{-}6h}$ = 0.0057 (**), $p_{Glu\text{-}24h}$ = 0.009 (**), $p_{Glu+BINA\text{-}2h}$ = 0.014 (*), $p_{Glu+BINA\text{-}4h}$ = 0.00025 (***), $p_{Glu+BINA\text{-}6h}$ = 0.00024 (***), $p_{Glu+BINA\text{-}24h}$ = 0.000008 (****), n = 3 independent biological samples examined over 3 independent experiments. Individual biological replicates are given together with the mean +/- SD.

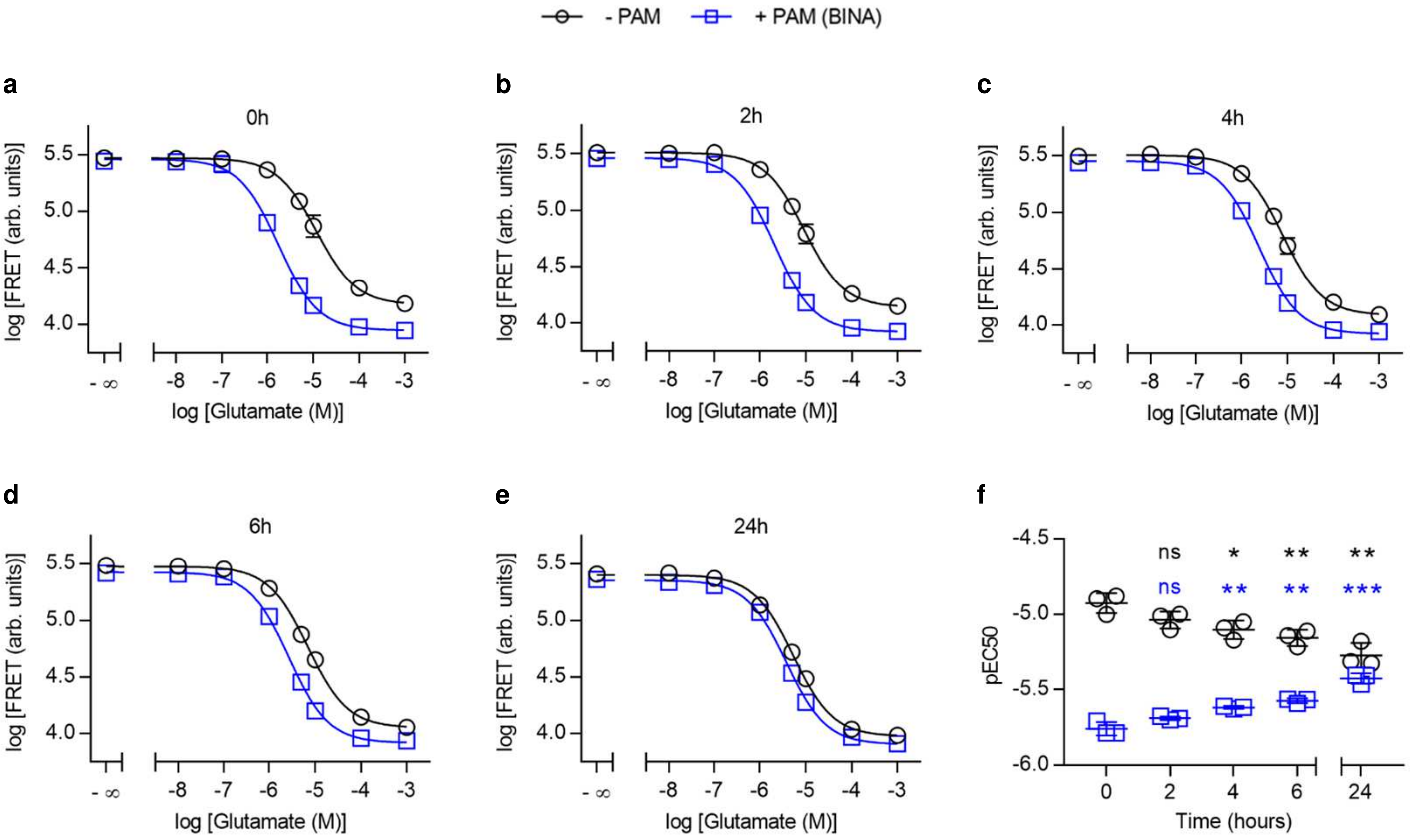

Supplementary Figure 5: Functional assessment of mGlu2 in 0.005% LMNG + 0.0002% CHS by LRET. a-e) Dose-response curves of Glutamate in the absence (- PAM, black circles) and presence of 10 µM BINA (+ PAM, blue squares) recorded after different time intervals of sample storage at room temperature. Data represent the mean of three biological replicates +/- SD, each measured in triplicates. f) Change of pEC_{50} values over time, obtained from dose-response curves in a-e. Statistical differences of pEC_{50} values for Glu (black) and Glu + BINA (blue) compared to time 0h were determined using two-sided unpaired t-tests and are given as p_{Glu-2h} = 0.089 (ns), p_{Glu-4h} = 0.028 (*), p_{Glu-6h} = 0.0094 (**), $p_{Glu-24h}$ = 0.0047 (**), $p_{Glu+BINA-2h}$ = 0.057 (ns), $p_{Glu+BINA-4h}$ = 0.0057 (**), $p_{Glu+BINA-6h}$ = 0.0024 (**), $p_{Glu+BINA-24h}$ = 0.00048 (***), n = 3 independent biological samples examined over 3 independent experiments. Individual biological replicates are given together with the mean +/- SD.

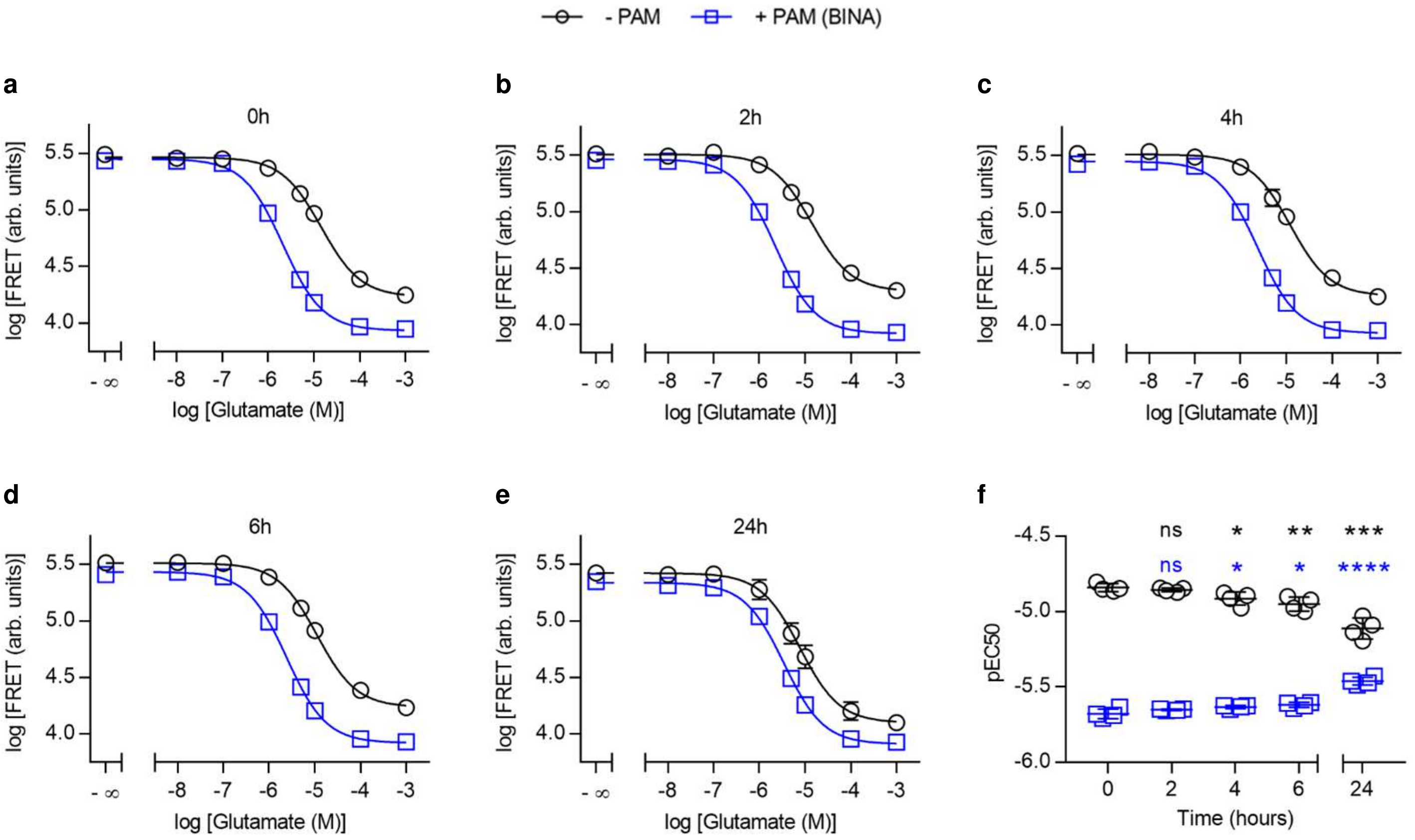

Supplementary Figure 6: Functional assessment of mGlu2 in 0.005% LMNG + 0.0004% CHS by LRET. a-e) Dose-response curves of Glutamate in the absence (- PAM, black circles) and presence of 10 µM BINA (+ PAM, blue squares) recorded after different time intervals of sample storage at room temperature. Data represent the mean of four biological replicates +/- SD, each measured in triplicates. f) Change of pEC_{50} values over time, obtained from dose-response curves in a-e. Statistical differences of pEC_{50} values for Glu (black) and Glu + BINA (blue) compared to time 0h were determined using two-sided unpaired t-tests and are given as $p_{Glu\text{-}2h}$ = 0.33 (ns), $p_{Glu\text{-}4h}$ = 0.027 (*), $p_{Glu\text{-}6h}$ = 0.0063 (**), $p_{Glu\text{-}24h}$ = 0.00035 (***), $p_{Glu+BINA\text{-}2h}$ = 0.16 (ns), $p_{Glu+BINA\text{-}4h}$ = 0.039 (*), $p_{Glu+BINA\text{-}6h}$ = 0.018 (*), $p_{Glu+BINA\text{-}24h}$ = 0.00004 (****); n = 4 independent biological samples examined over 1 independent experiment. Individual biological replicates are given together with the mean +/- SD.

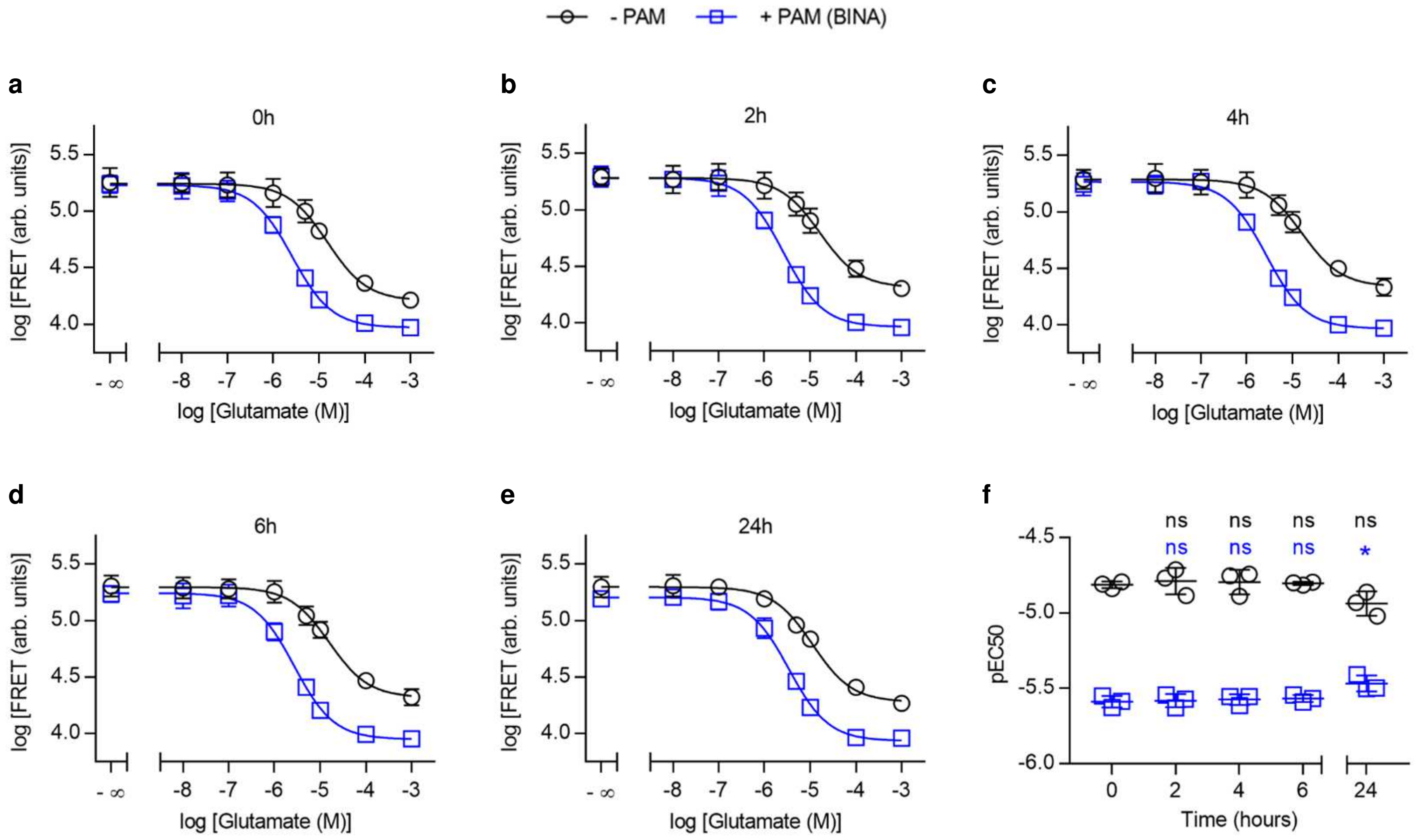

Supplementary Figure 7: Functional assessment of mGlu2 in 0.005% LMNG + 0.0008% CHS by LRET. a-e) Dose-response curves of Glutamate in the absence (- PAM, black circles) and presence of 10 µM BINA (+ PAM, blue squares) recorded after different time intervals of sample storage at room temperature. Data represent the mean of three biological replicates +/- SD, each measured in triplicates. f) Change of pEC_{50} values over time, obtained from dose-response curves in a-e. Statistical differences of pEC_{50} values for Glu (black) and Glu + BINA (blue) compared to time 0h were determined using two-sided unpaired t-tests and are given as $p_{Glu\text{-}2h} = 0.66$ (ns), $p_{Glu\text{-}4h} = 0.73$ (ns), $p_{Glu\text{-}6h} = 0.51$ (ns), $p_{Glu\text{-}24h} = 0.06$ (ns), $p_{Glu+BINA\text{-}2h} = 0.86$ (ns), $p_{Glu+BINA\text{-}4h} = 0.68$ (ns), $p_{Glu+BINA\text{-}6h} = 0.47$ (ns), $p_{Glu+BINA\text{-}24h} = 0.034$ (*); n = 3 independent biological samples examined over 3 independent experiments. Individual biological replicates are given together with the mean +/- SD.

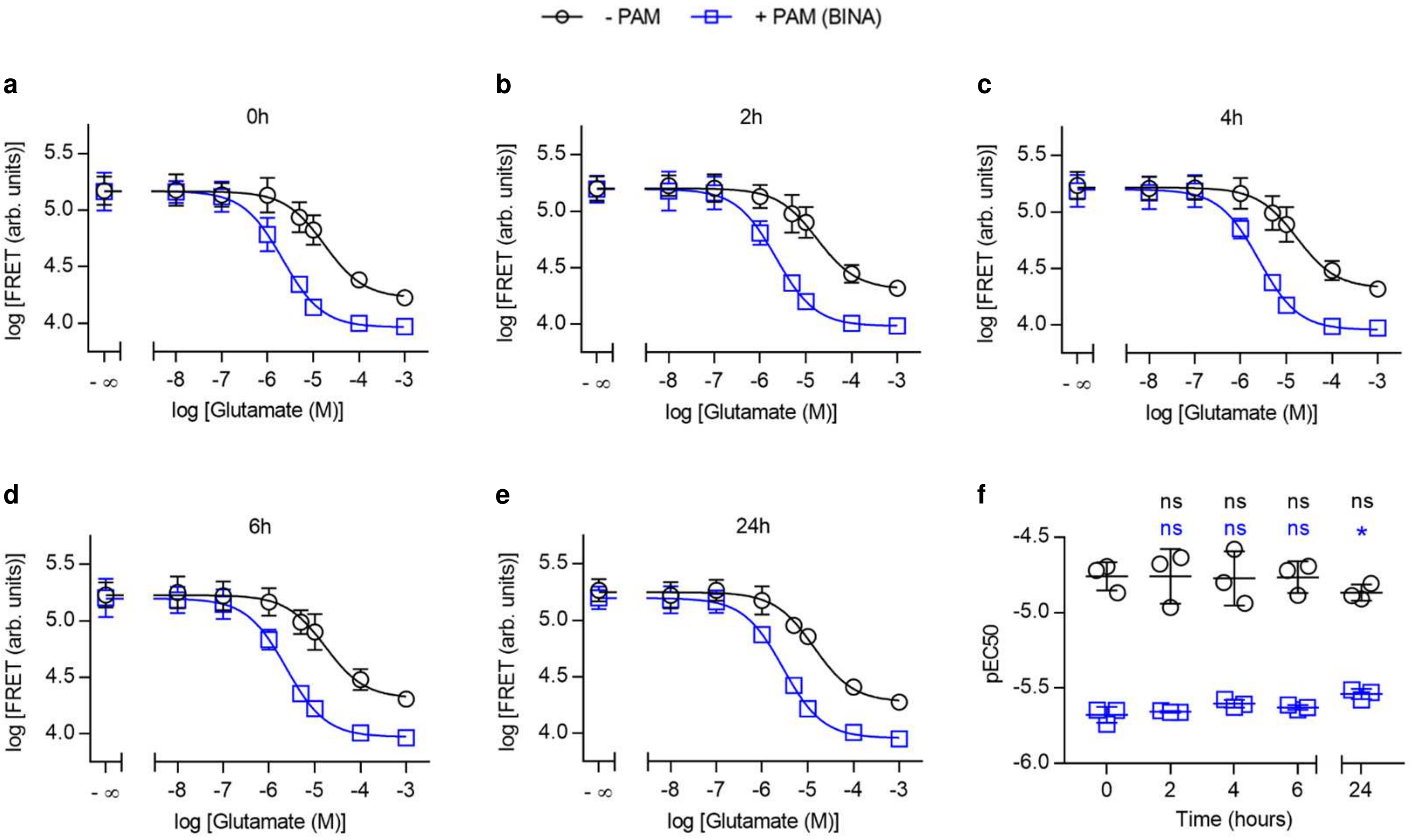

Supplementary Figure 8: Functional assessment of mGlu2 in 0.005% LMNG + 0.0004% CHS + 0.0025% GDN by LRET. a-e) Dose-response curves of Glutamate in the absence (- PAM, black circles) and presence of 10 µM BINA (+ PAM, blue squares) recorded after different time intervals of sample storage at room temperature. (+ PAM, blue squares) recorded after different time intervals of sample storage at room temperature. Data represent the mean of three biological replicates +/- SD, each measured in triplicates. f) Change of pEC_{50} values over time, obtained from dose-response curves in a-e. Statistical differences of pEC_{50} values for Glu (black) and Glu + BINA (blue) compared to time 0h were determined using two-sided unpaired t-tests and are given as $p_{Glu\text{-}2h}$ = 0.99 (ns), $p_{Glu\text{-}4h}$ = 0.93 (ns), $p_{Glu\text{-}6h}$ = 0.95 (ns), $p_{Glu\text{-}24h}$ = 0.16 (ns), $p_{Glu+BINA\text{-}2h}$ = 0.54 (ns), $p_{Glu+BINA\text{-}4h}$ = 0.099 (ns), $p_{Glu+BINA\text{-}6h}$ = 0.2 (ns), $p_{Glu+BINA\text{-}24h}$ = 0.02 (*); n = 3 independent biological samples examined over 3 independent experiments. Individual biological replicates are given together with the mean +/- SD.

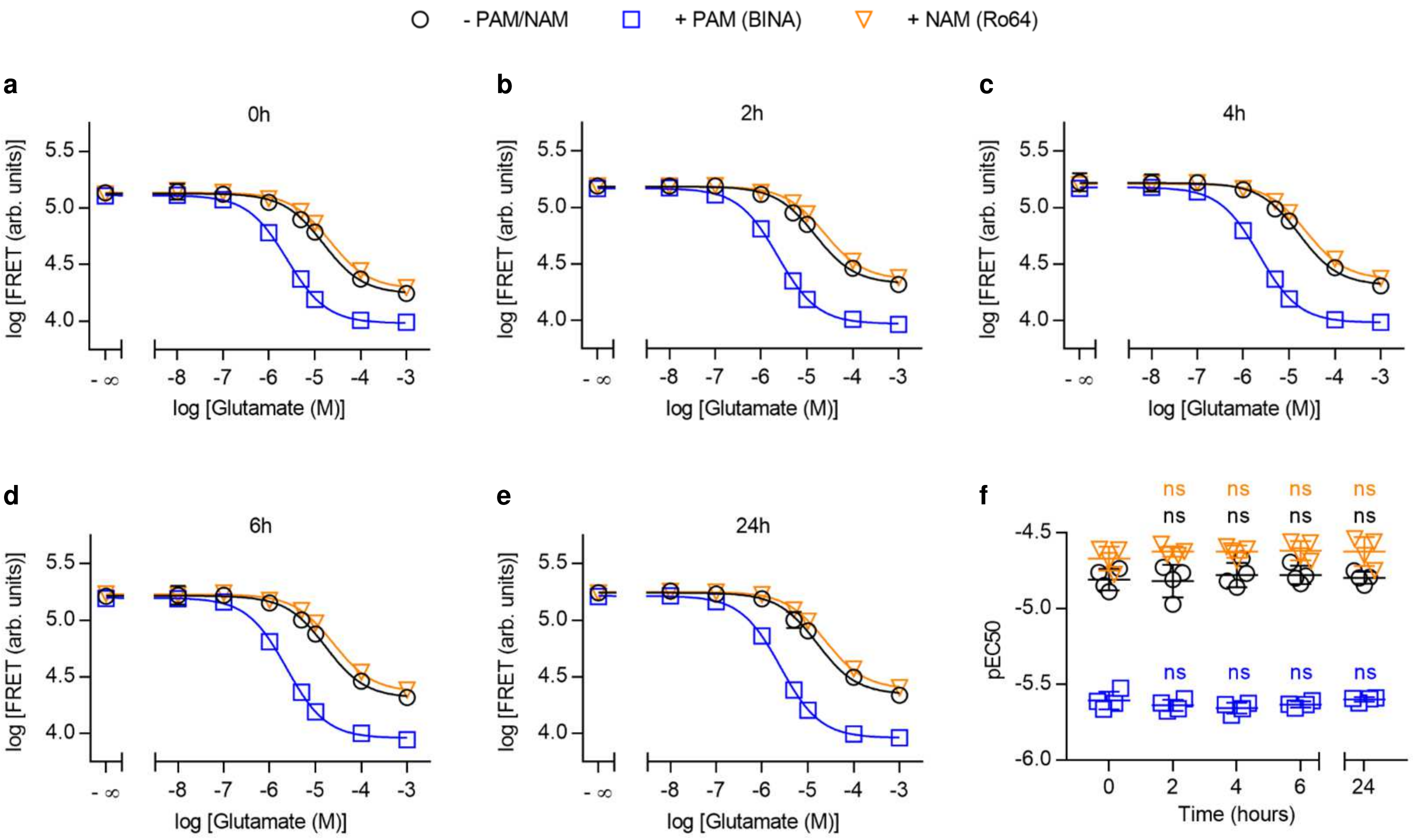

Supplementary Figure 9: Functional assessment of mGlu2 in 0.005% LMNG + 0.0004% CHS + 0.005% GDN by LRET. a-e) Dose-response curves of Glutamate in the absence (No Modulation, black circles) and presence of 10 µM BINA (+ BINA, blue squares) or 10 µM Ro64 (+ Ro64, orange triangles) recorded after different time intervals of sample storage at room temperature. Data represent the mean of four biological replicates +/- SD, each measured in triplicates. f) Change of pEC_{50} values over time, obtained from dose-response curves in a-e. Statistical differences of pEC_{50} values for Glu (black) and Glu + BINA (blue) compared to time 0h were determined using two-sided unpaired t-tests and are given as p_{Glu-2h} = 0.89 (ns), p_{Glu-4h} = 0.6 (ns), p_{Glu-6h} = 0.54 (ns), $p_{Glu-24h}$ = 0.78 (ns), $p_{Glu+BINA-2h}$ = 0.39 (ns), $p_{Glu+BINA-4h}$ = 0.2 (ns), $p_{Glu+BINA-6h}$ = 0.43 (ns), $p_{Glu+BINA-24h}$ = 0.83 (ns), $p_{Glu+Ro64-2h}$ = 0.32 (ns), $p_{Glu+BINA-4h}$ = 0.34 (ns), $p_{Glu+BINA-6h}$ = 0.33 (ns), $p_{Glu+BINA-24h}$ = 0.49 (ns); n = 4 independent biological samples examined over 3 independent experiments. Individual biological replicates are given together with the mean +/- SD.

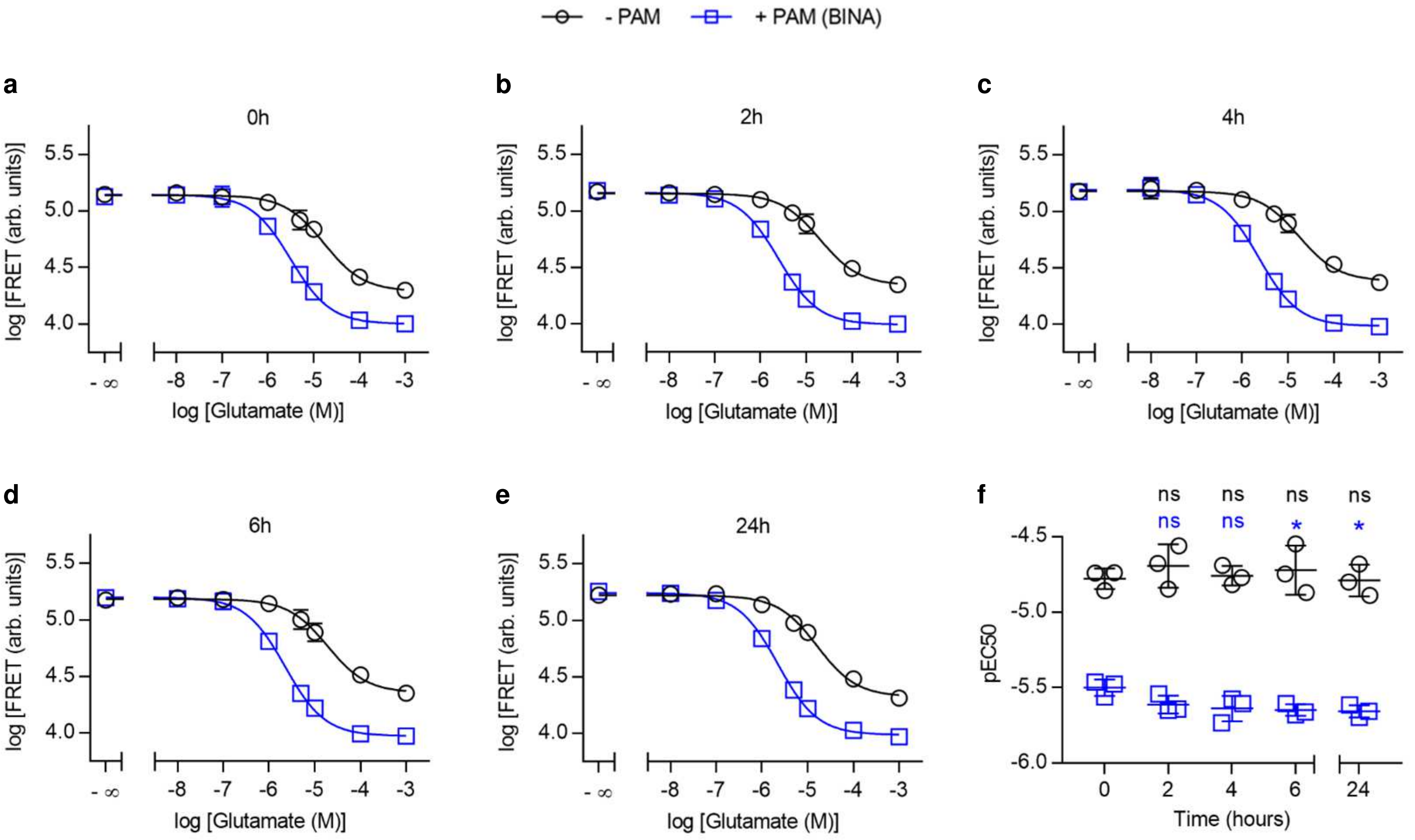

Supplementary Figure 10: Functional assessment of mGlu2 in 0.005% LMNG + 0.0004% CHS + 0.01% GDN by LRET. a-e) Dose-response curves of Glutamate in the absence (- PAM, black circles) and presence of 10 µM BINA (+ PAM, blue squares) recorded after different time intervals of sample storage at room temperature. Data represent the mean of three biological replicates +/- SD, each measured in triplicates. f) Change of pEC_{50} values over time, obtained from dose-response curves in a-e. Statistical differences of pEC_{50} values for Glu (black) and Glu + BINA (blue) compared to time 0h were determined using two-sided unpaired t-tests and are given as p_{Glu-2h} = 0.41 (ns), p_{Glu-4h} = 0.74 (ns), p_{Glu-6h} = 0.61 (ns), $p_{Glu-24h}$ = 0.88 (ns), $p_{Glu+BINA-2h}$ = 0.074 (ns), $p_{Glu+BINA-4h}$ = 0.074 (ns), $p_{Glu+BINA-6h}$ = 0.018 (*), $p_{Glu+BINA-24h}$ = 0.016 (*); n = 3 independent biological samples examined over 3 independent experiments. Individual biological replicates are given together with the mean +/- SD.

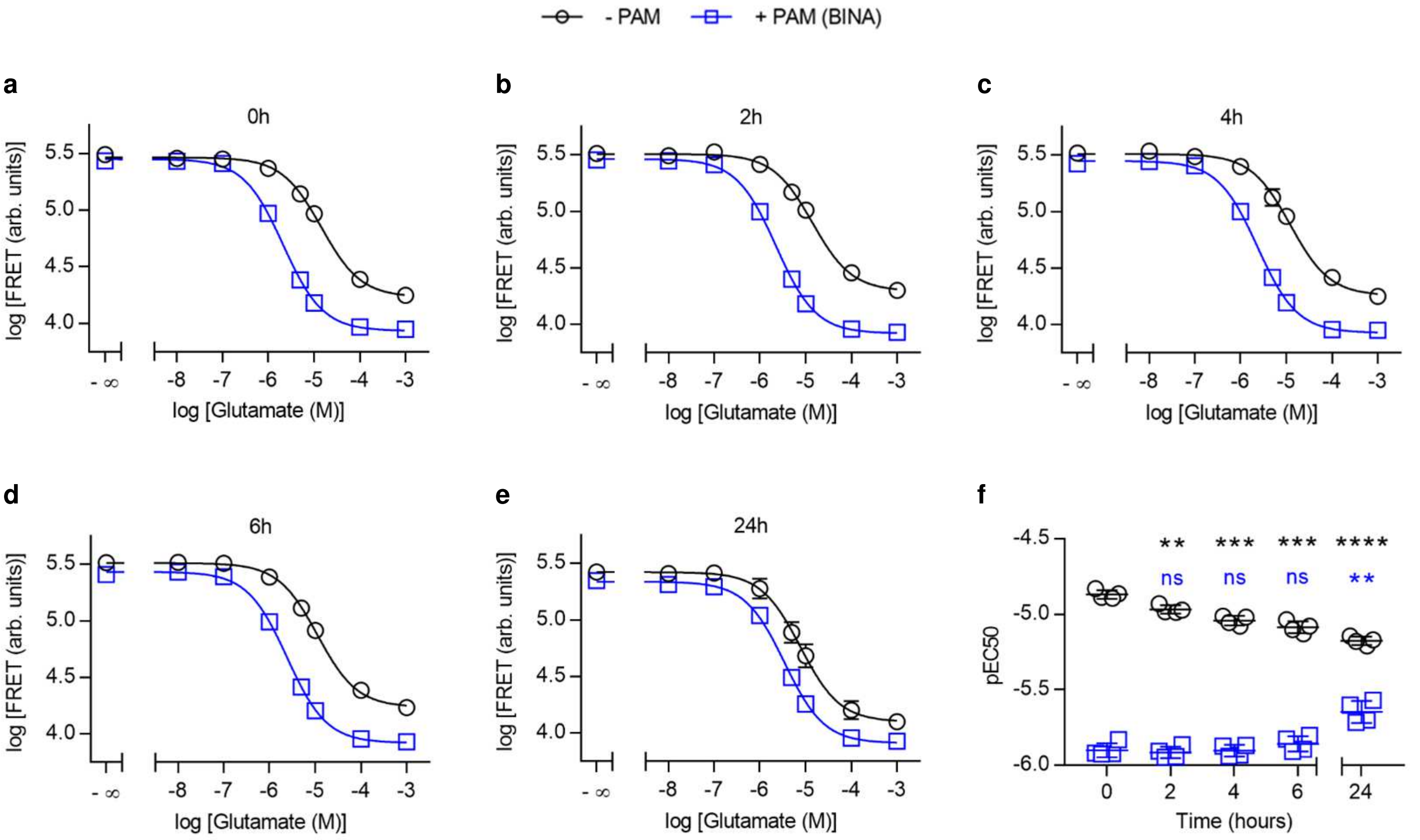

Supplementary Figure 11: Functional assessment of mGlu2 in 0.005% LMNG + 0.005% GDN by LRET. a-e) Dose-response curves of Glutamate in the absence (- PAM, black circles) and presence of 10 µM BINA (+ PAM, blue squares) recorded after different time intervals of sample storage at room temperature. Data represent the mean of four biological replicates +/- SD, each measured in triplicates. f) Change of pEC_{50} values over time, obtained from dose-response curves in a-e. Statistical differences of pEC_{50} values for Glu (black) and Glu + BINA (blue) compared to time 0h were determined using two-sided unpaired t-tests and are given as p_{Glu-2h} = 0.0025 (**), p_{Glu-4h} = 0.000199 (***), p_{Glu-6h} = 0.00012 (***), $p_{Glu-24h}$ = 0.000005 (****), $p_{Glu+BINA-2h}$ = 0.63 (ns), $p_{Glu+BINA-4h}$ = 0.91 (ns), $p_{Glu+BINA-6h}$ = 0.27 (ns), $p_{Glu+BINA-24h}$ = 0.0011 (**); n = 4 independent biological samples examined over 1 independent experiments. Individual biological replicates are given together with the mean +/- SD.

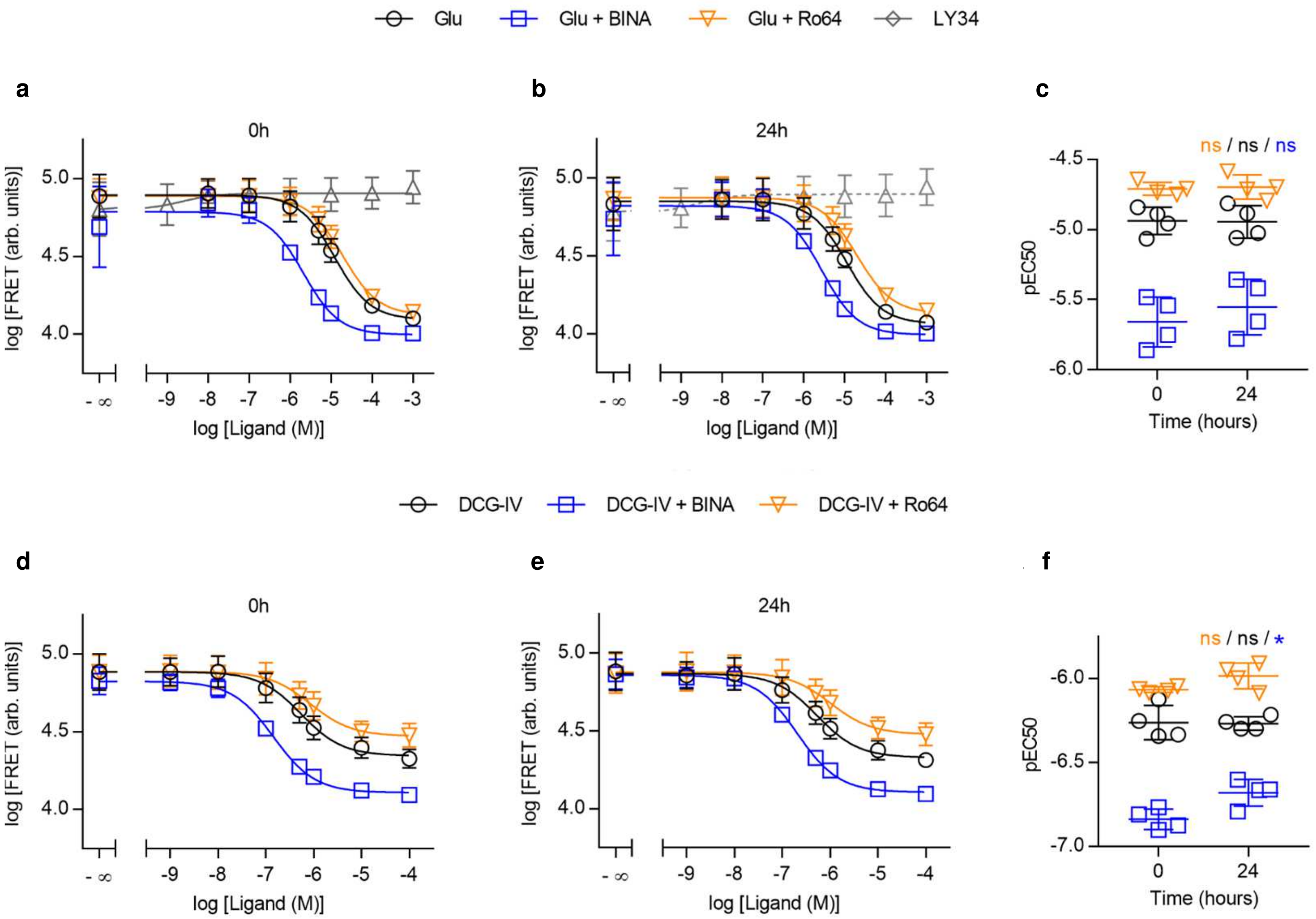

Supplementary Figure 12: Orthosteric ligand response of mGlu2 in membranes by LRET. a-b) Dose-response curves of LY34 alone (grey triangles) and Glutamate in the absence (black circles) and presence of 10 µM BINA (blue squares) or 10 µM Ro64 (orange triangles) recorded after different time intervals of sample storage at room temperature. Data represent the mean of four biological replicates +/- SD, each measured in triplicates. c) Change of pEC_{50} values over time, obtained from dose-response curves in a-b. d-e) Dose-response curves of DCG-IV in the absence (black circles) and presence of 10 µM BINA (blue squares) or 10 µM Ro64 (orange triangles) recorded after different time intervals of sample storage at room temperature. Data represent the mean of four biological replicates +/- SD, each measured in triplicates. f) Change of pEC_{50} values over time, obtained from dose-response curves in d-e. Statistical differences of pEC_{50} values for Glu + Ro64 or DCG-IV + Ro64 (yellow), Glu or DCG-IV (black) and Glu + BINA or DCG-IV + BINA (blue) compared to time 0h were determined using two-sided, unpaired t-tests and are given as $p_{Glu-24h}$ = 0.92 (ns), $p_{Glu+BINA-24h}$ = 0.46 (ns); $p_{Glu+Ro64-24h}$ = 0.8 (ns); $p_{DCG-IV-24h}$ = 0.93 (ns); $p_{DCG-IV+BINA-24h}$ = 0.02 (ns); $p_{DCG-IV+Ro64-24h}$ =0.084 (*); n = 4 independent biological samples examined over 3 independent experiments.

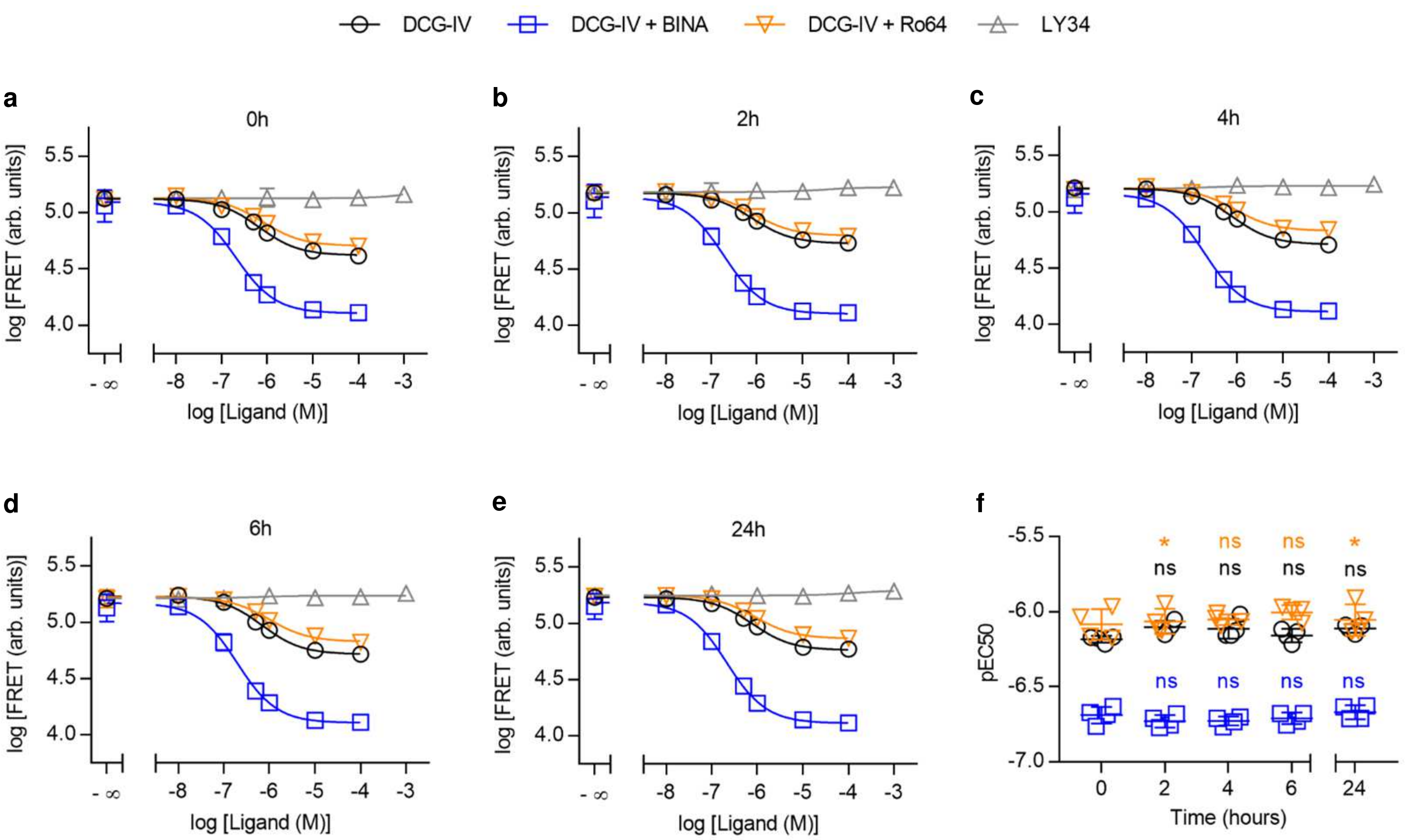

Supplementary Figure 13: Orthosteric ligand response of mGlu2 in 0.005% LMNG + 0.0004% CHS + 0.005% GDN by LRET. a-e) Dose-response curves of LY34 alone (grey triangles) and DCG-IV in the absence (black circles) and presence of 10 µM BINA (blue squares) or 10 µM Ro64 (orange triangles) recorded after different time intervals of sample storage at room temperature. Data represent the mean of four biological replicates +/- SD, each measured in triplicates. f) Change of pEC_{50} values over time, obtained from dose-response curves in a-e. Statistical differences of pEC_{50} values for DCG-IV (black) and DCG-IV + BINA (blue) compared to time 0h were determined using two-sided unpaired t-tests and are given as $p_{DCG\text{-}IV\text{-}2h}$ = 0.017 (ns), $p_{DCG\text{-}IV\text{-}4h}$ = 0.095 (ns), $p_{DCG\text{-}IV\text{-}6h}$ = 0.35 (ns), $p_{DCG\text{-}IV\text{-}24h}$ = 0.0072 (ns), $p_{DCG\text{-}IV+BINA\text{-}2h}$ = 0.27 (ns), $p_{DCG\text{-}IV+BINA\text{-}4h}$ = 0.25 (ns), $p_{DCG\text{-}IV+BINA\text{-}6h}$ = 0.55 (ns), $p_{DCG\text{-}IV+BINA\text{-}24h}$ = 0.64 (ns), $p_{DCG\text{-}IV+Ro64\text{-}2h}$ = 0.77 (*), $p_{DCG\text{-}IV+BINA\text{-}4h}$ = 0.58 (ns), $p_{DCG\text{-}IV+BINA\text{-}6h}$ = 0.21 (ns), $p_{DCG\text{-}IV+BINA\text{-}24h}$ = 0.7 (*); n = 4 independent biological samples examined over 3 independent experiments. Individual biological replicates are given together with the mean +/- SD.

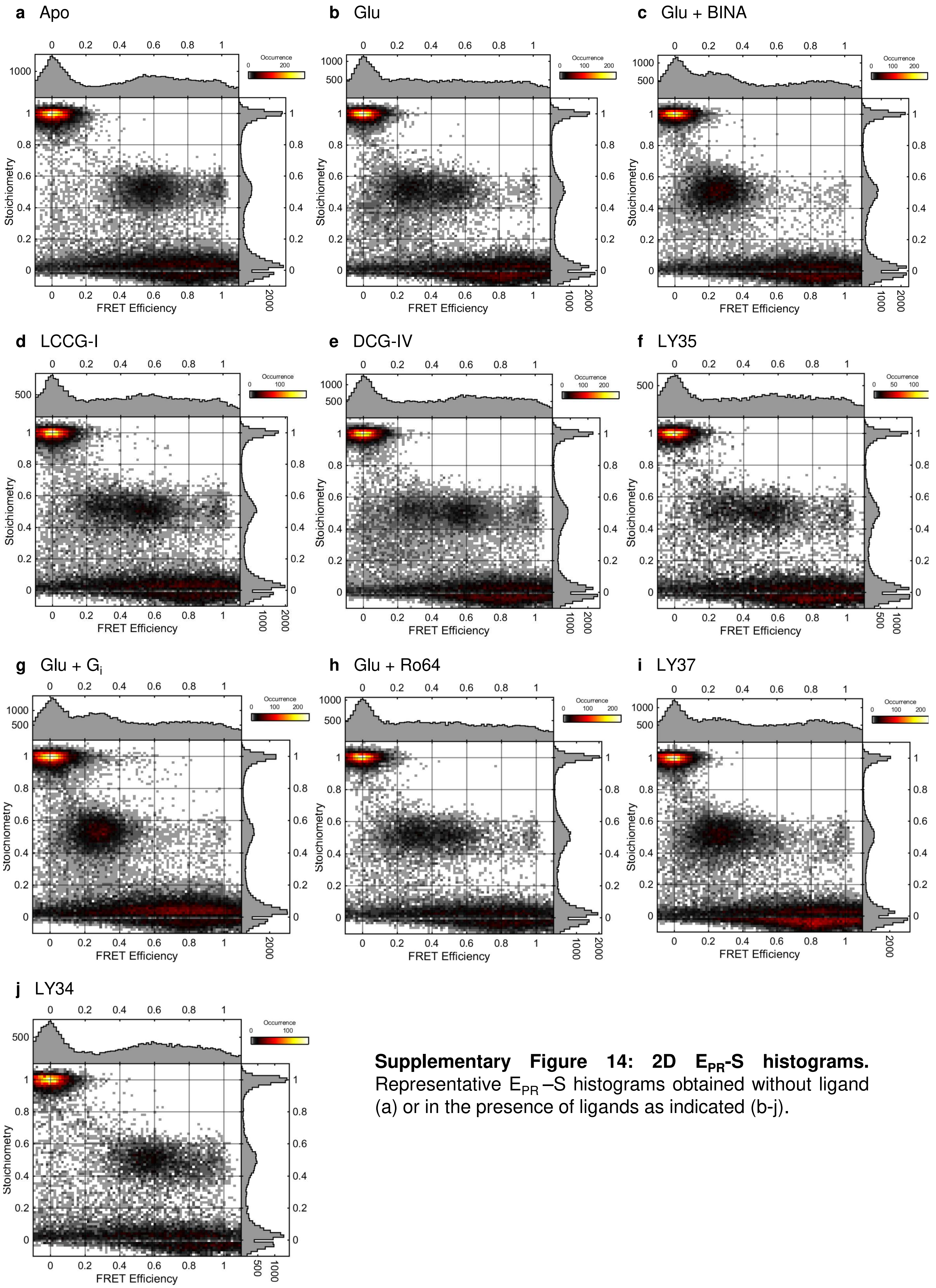

Supplementary Figure 14: 2D E_{PR}-S histograms. Representative E_{PR}–S histograms obtained without ligand (a) or in the presence of ligands as indicated (b-j).

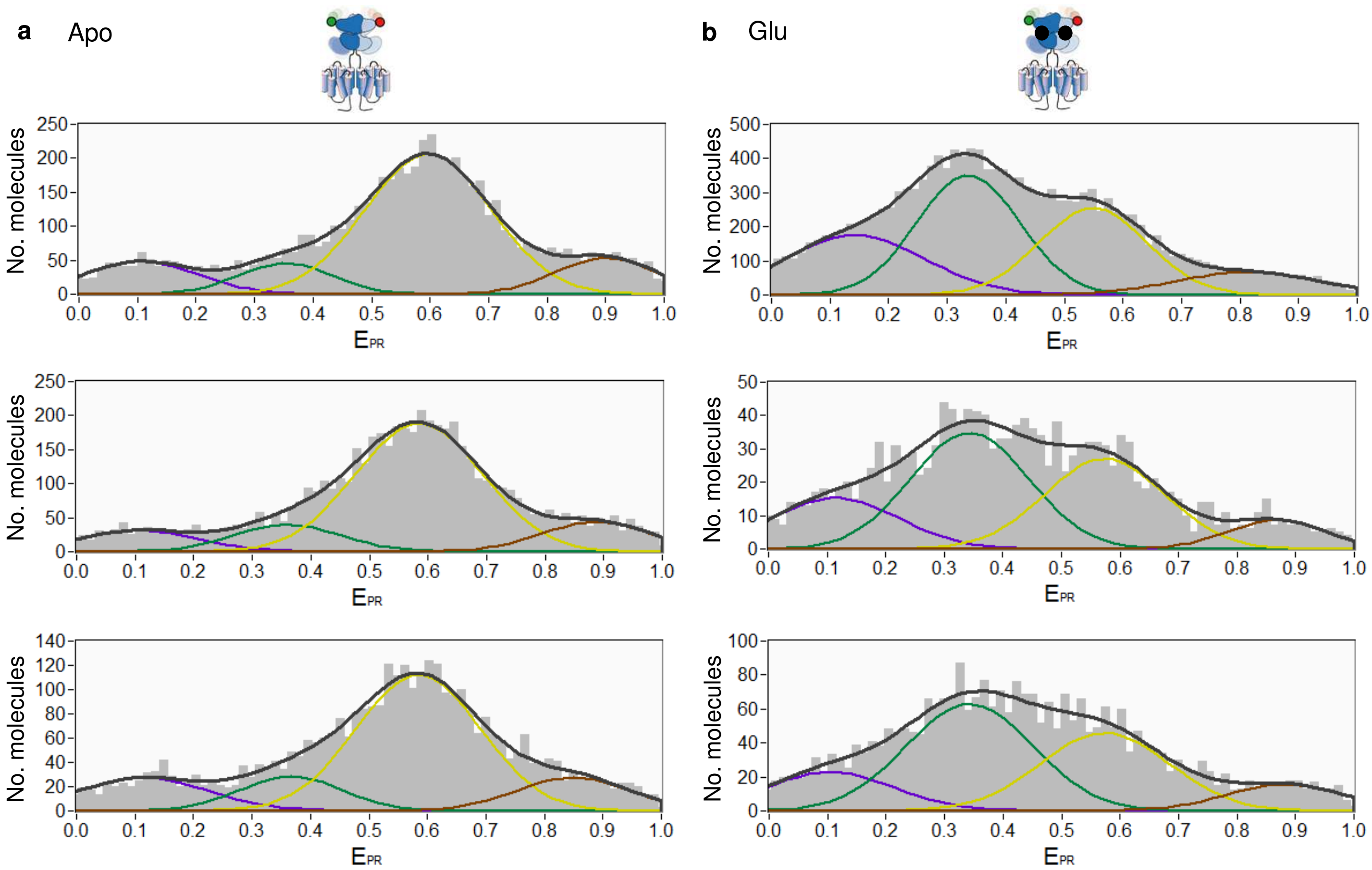

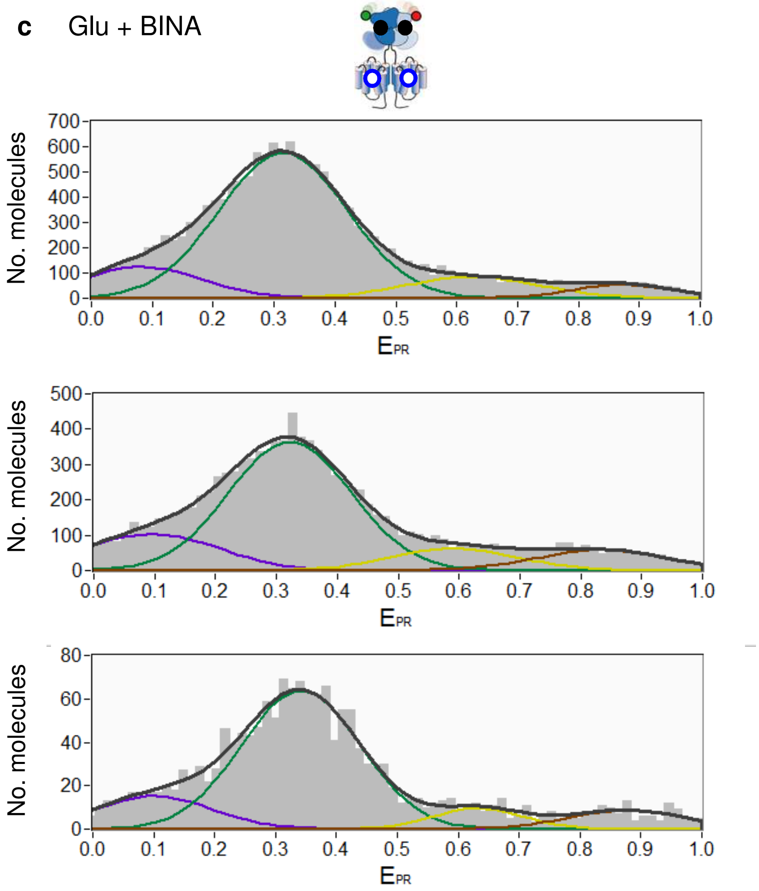

Supplementary Figure 15: Representative FRET histograms showing the reproducibility of the experiments. smFRET histograms obtained without ligand (a), or in the presence of Glu (b), and Glu + BINA (c). Each row of histograms represents an independent set of experiments of a biological replicate of solubilized receptors originating from membrane fractions prepared at different cell passages.

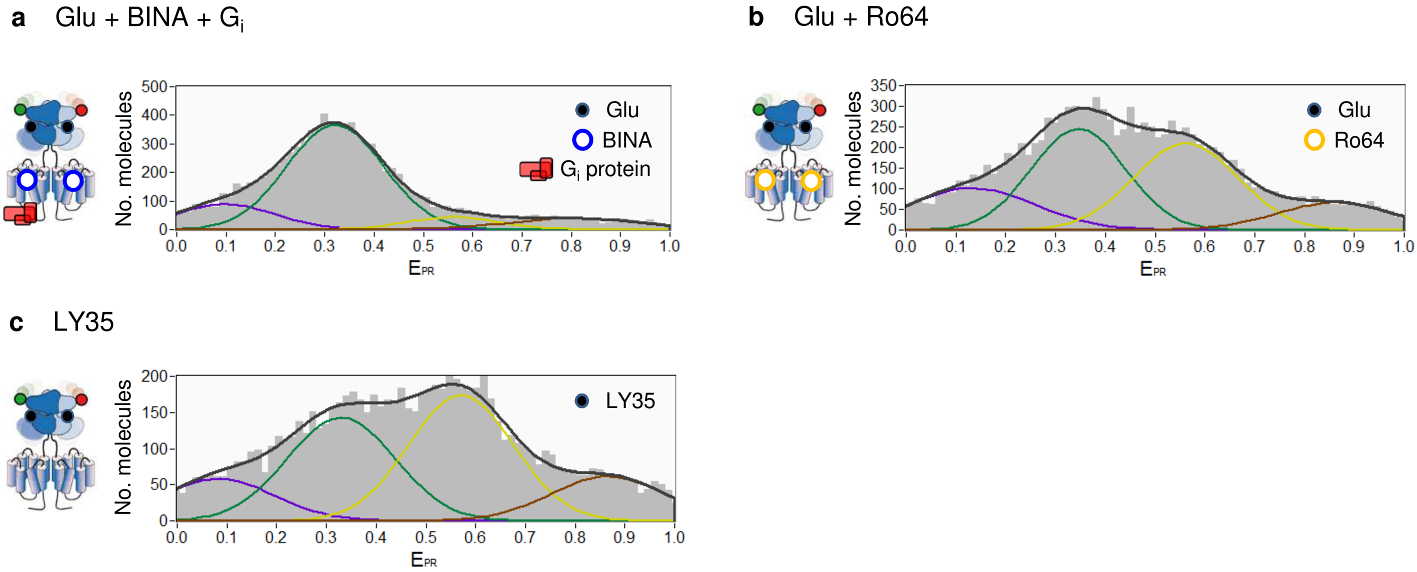

Supplementary Figure 16: FRET histograms for additional ligands. Representative E_{PR} histograms with Glu + BINA + G_i (a), Glu + Ro64 (b), LY35 (c). FRET distributions were fitted with four gaussians alternatingly keeping either E_{PR} or FWHM variable until no further improvement was achieved. The sum of the four gaussians is displayed in black.

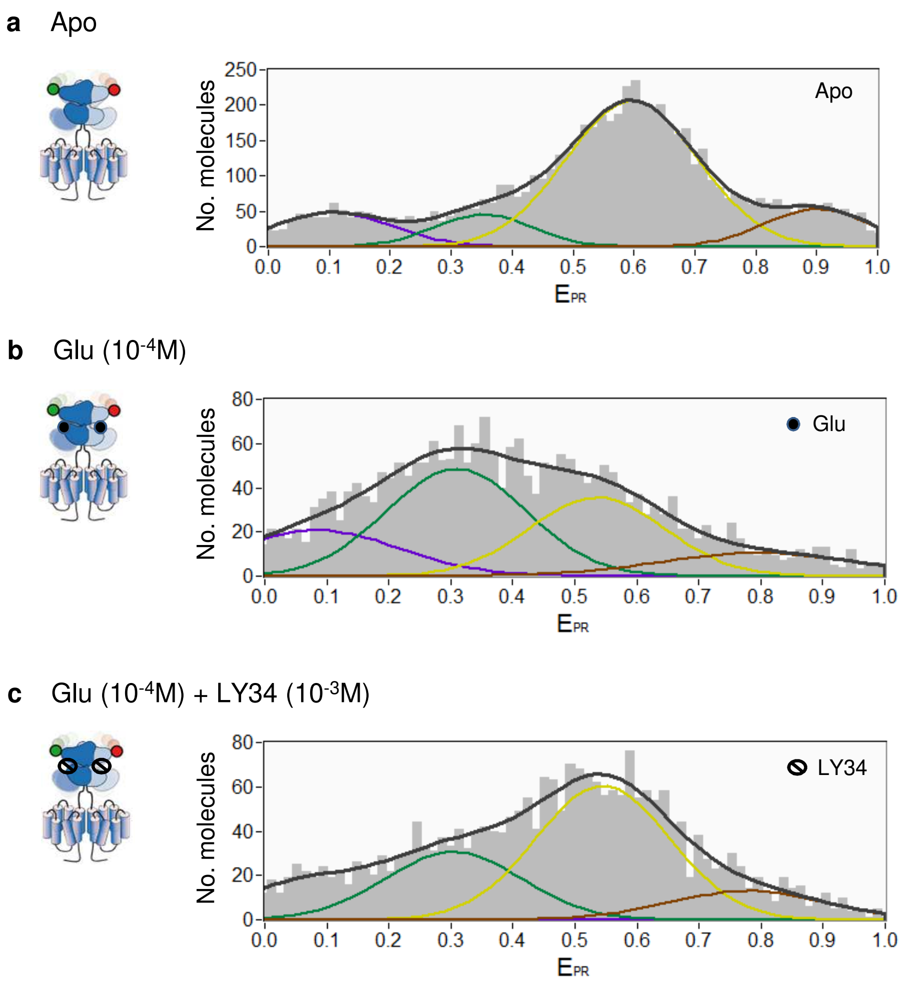

Supplementary Figure 17: FRET histograms showing the reversal of glutamate effect by the competitive orthosteric antagonist LY34. Representative E_{PR} histograms without ligand (a), or in the presence of Glu (10^{-4}M) (b), and Glu + LY34 (10^{-3}M) (c). FRET distributions were fitted with four Gaussians alternatingly keeping either E_{PR} or FWHM variable until no further improvement was achieved. The sum of the four Gaussians is displayed in black.

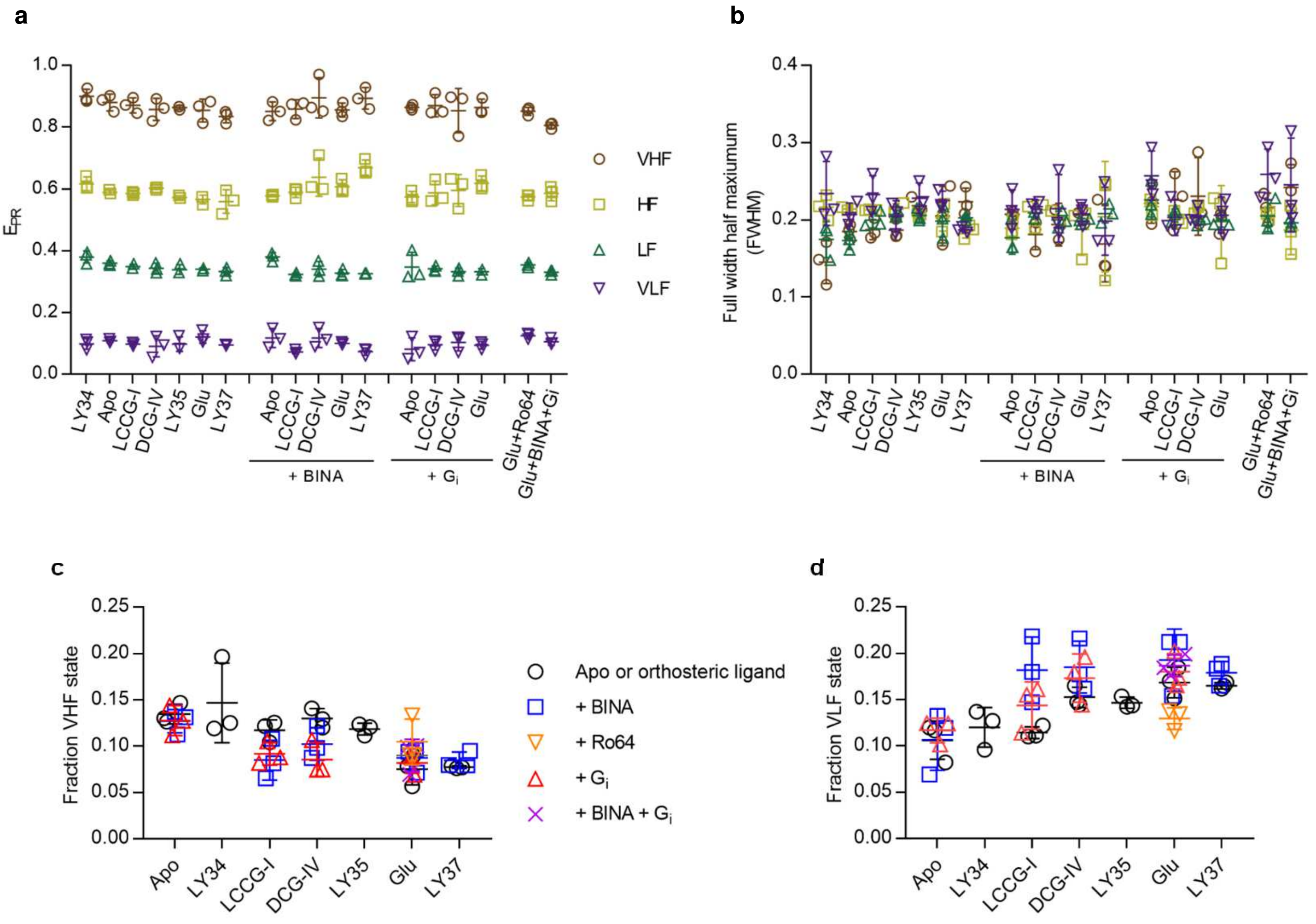

Supplementary Figure 18: Determination of average FRET, FWHM and fraction of VHF and VLF states. a-b) FRET distributions were fitted with four gaussians alternatingly keeping either E_{PR} or full width half maxiumum (FWHM) variable until no further improvement was achieved. c-d) Fraction of VHF and VLF at fixed mean E_{PR} (VLF: 0.1, LF: 0.34, HF: 0.6, VHF: 0.87) and FWHM (0.2) at various ligand conditions. Data are given as the mean of three biological replicates with errors given as standard deviation.

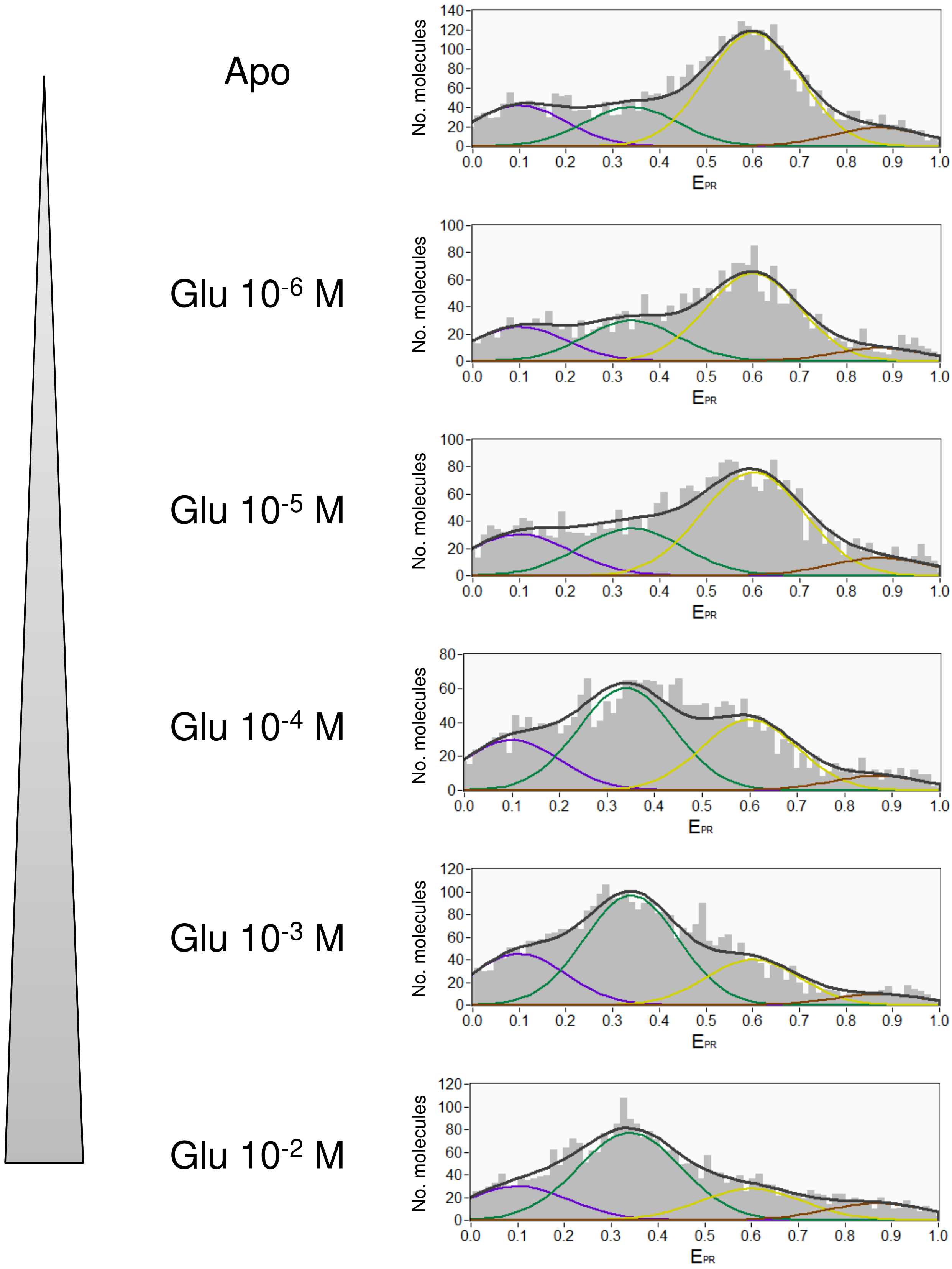

Supplementary Figure 19: Representative FRET histograms of a titration of mGlu2 with increasing concentrations of glutamate. Such data were used to generate the titration curves presented in Figure 3i. FRET distributions were fitted at fixed mean E_{PR} (VLF: 0.1, LF: 0.34, HF: 0.6, VHF: 0.87) and FWHM (0.2) . The sum of the four gaussians is displayed in black.

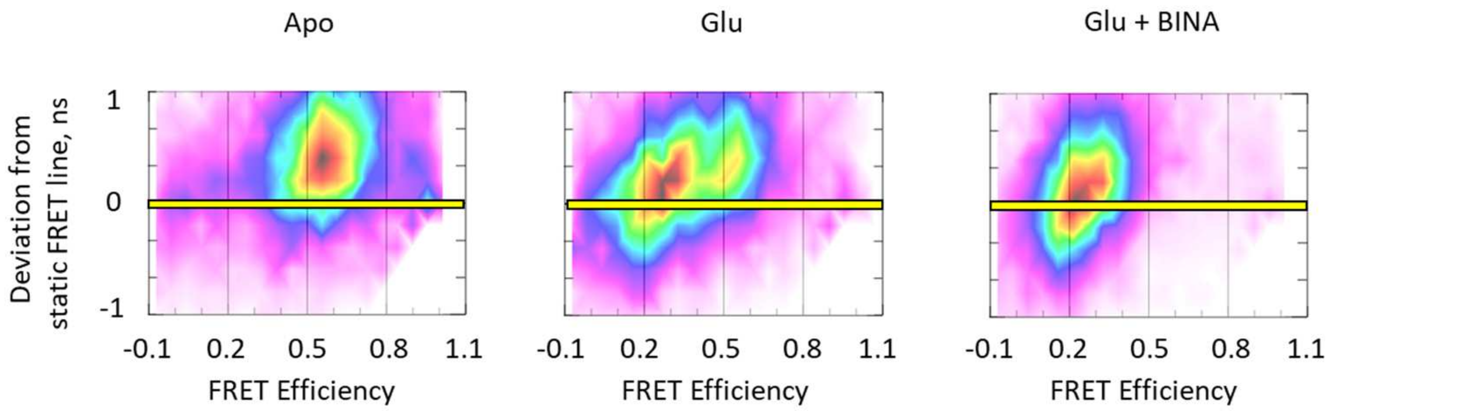

Supplementary Figure 20: Alternative representation of the τ_{DA} vs. *E* histograms presented in Figure 4a-c. The "static FRET line" is represented horizontally (yellow) and the y axis shows the deviation from this line (in ns). For the Apo receptors, the major population appears above the "static FRET line" indicating conformational dynamics at the sub-millisecond time scale. The combined addition of Glu and BINA leads to a displacement of the population to the center of the "static FRET line", indicating a conformational stabilization of the receptors.

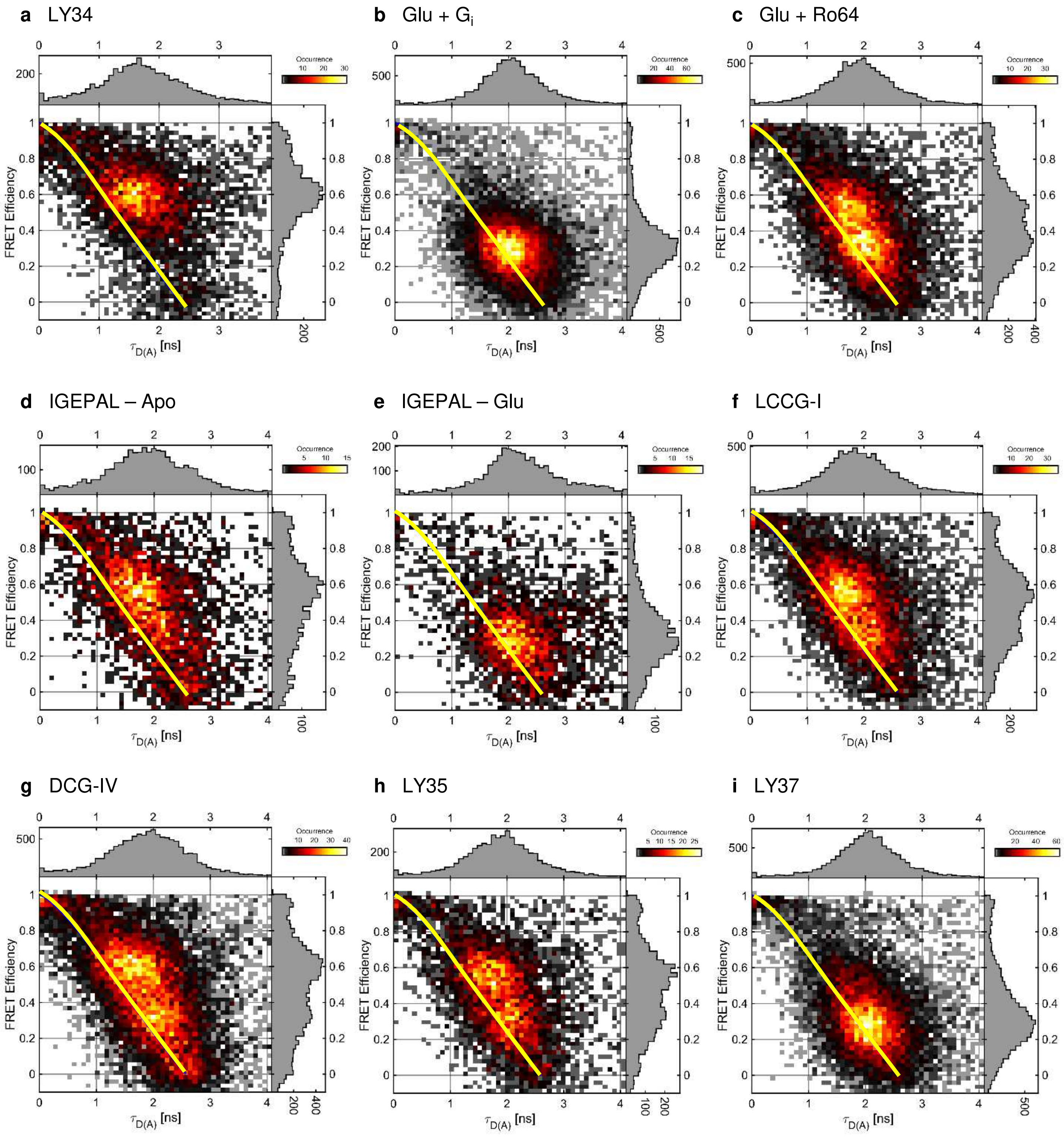

Supplementary Figure 21: τ_{DA} vs *E* histogram under additional ligand conditions. Representative τ_{DA} vs *E* histograms obtained in the presence of antagonist LY34 (a), Glu + G_i (b), Glu + Ro64 (c), partial agonists LCCG-I (f), DCG-IV (g), LY35 (h), superagonist LY37 (i), in LMNG-CHS-GDN and in the absence (d) or presence of Glu (e) in IGEPAL. The yellow line represents the theoretical "static FRET line".

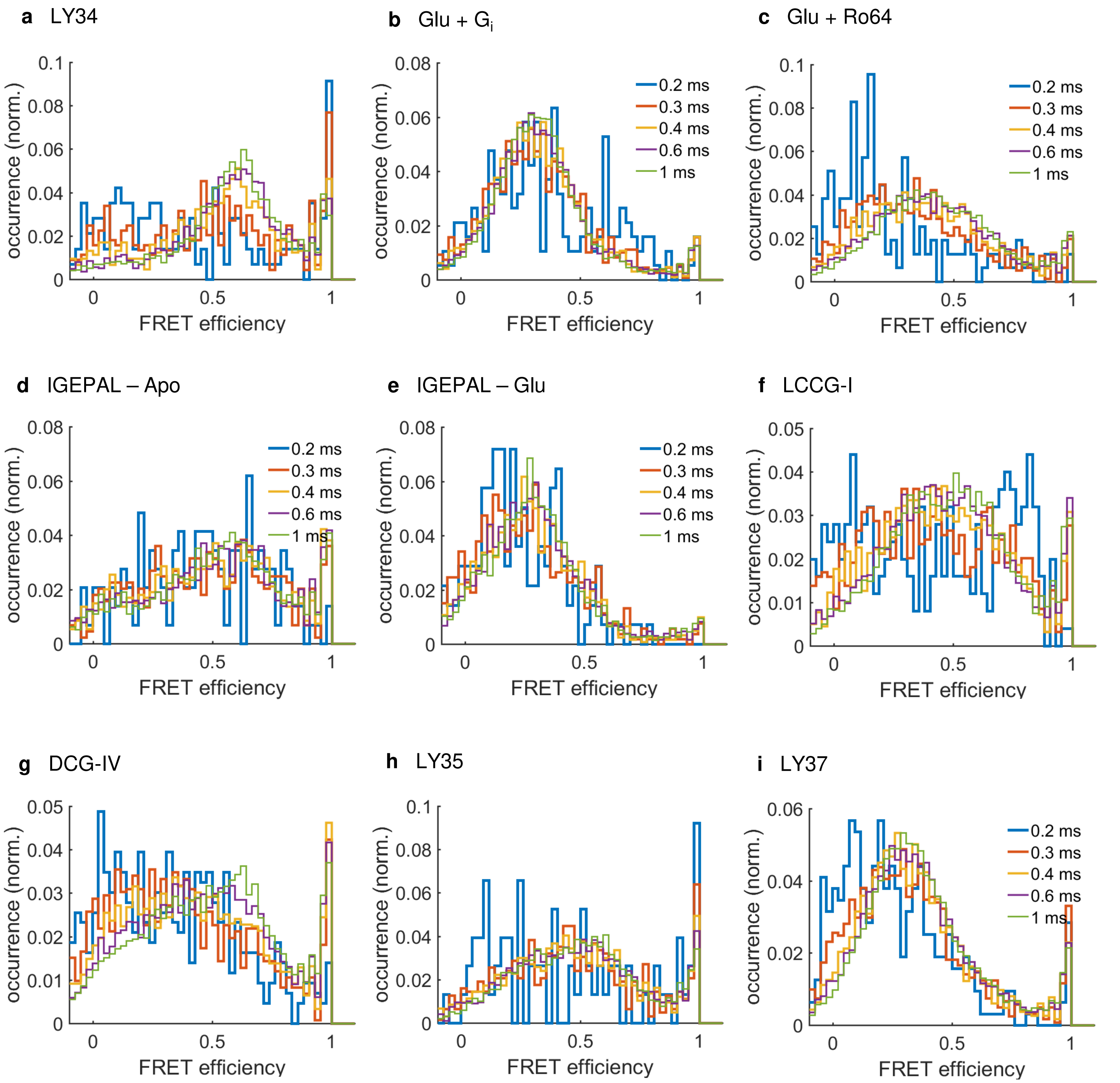

Supplementary Figure 22: Time window analysis (TWA) under additional conditions. Representative TWA histograms at 0.2 to 1 ms integration times in the presence of antagonist LY34 (a), Glu + G_i (b), Glu + Ro64 (c), partial agonists LCCG-I (f), DCG-IV (g), LY35 (h), superagonist LY37 (i), in LMNG-CHS-GDN and in the absence (d) or presence of Glu (e) in IGEPAL.

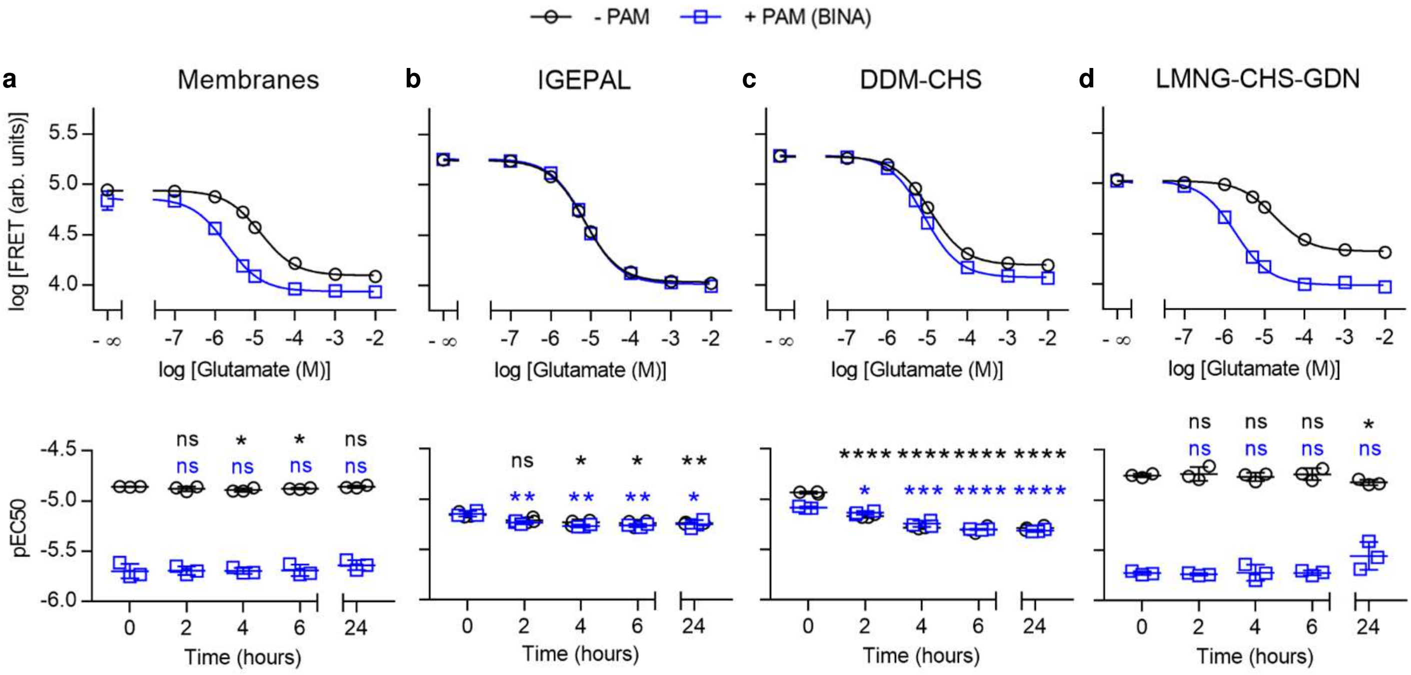

Supplementary Figure 23: Functional assessment of rat SNAP-mGlu2 by LRET. Top row) Dose-response curves of Glutamate in the absence (- PAM, black circles) and presence of 10 µM BINA (+ PAM, blue squares) recorded at time t = 0 hours in membranes (a), IGEPAL (b), DDM-CHS (c) and LMNG-CHS-GDN (d). Bottom row) Corresponding changes of pEC_{50} values over time after storage at room temperature, obtained from dose-response curves. Data represent the mean of three biological replicates +/- SD, each measured in triplicates. Statistical differences of pEC_{50} values for Glu (black) and Glu + BINA (blue) compared to time 0h were determined using two-sided unpaired t-tests and are given as: a) $p_{Glu\text{-}2h}$ = 0.24 (ns), $p_{Glu\text{-}4h}$ = 0.045 (*), $p_{Glu\text{-}6h}$ = 0.021 (*), $p_{Glu\text{-}24h}$ = 0.97 (ns), $p_{Glu+BINA\text{-}2h}$ = 0.91 (ns), $p_{Glu+BINA\text{-}4h}$ = 0.95 (ns), $p_{Glu+BINA\text{-}6h}$ = 0.9 (ns), $p_{Glu+BINA\text{-}24h}$ = 0.3 (ns); b) $p_{Glu\text{-}2h}$ = 0.07 (ns), $p_{Glu\text{-}4h}$ = 0.031 (*), $p_{Glu\text{-}6h}$ = 0.032 (*), $p_{Glu\text{-}24h}$ = 0.0073 (**), $p_{Glu+BINA\text{-}2h}$ = 0.0086 (**), $p_{Glu+BINA\text{-}4h}$ = 0.002 (**), $p_{Glu+BINA\text{-}6h}$ = 0.0028 (**), $p_{Glu+BINA\text{-}24h}$ = 0.023 (*); c) $p_{Glu\text{-}2h}$ = 0.00004 (****), $p_{Glu\text{-}4h}$ = 0.000004 (****), $p_{Glu\text{-}6h}$ = 0.000079 (****), $p_{Glu\text{-}24h}$ = 0.000023 (****), $p_{Glu+BINA\text{-}2h}$ = 0.012 (*), $p_{Glu+BINA\text{-}4h}$ = 0.0009 (***), $p_{Glu+BINA\text{-}6h}$ = 0.000007 (****), $p_{Glu+BINA\text{-}24h}$ = 0.000013 (****); d) $p_{Glu\text{-}2h}$ = 0.72 (ns), $p_{Glu\text{-}4h}$ = 0.64 (ns), $p_{Glu\text{-}6h}$ = 0.75 (ns), $p_{Glu\text{-}24h}$ = 0.023 (*), $p_{Glu+BINA\text{-}2h}$ = 0.4 (ns), $p_{Glu+BINA\text{-}4h}$ = 0.92 (ns), $p_{Glu+BINA\text{-}6h}$ = 0.9 (ns), $p_{Glu+BINA\text{-}24h}$ = 0.1 (ns); n = 3 independent biological samples examined over 1 independent experiments. Individual biological replicates are given together with the mean +/- SD.

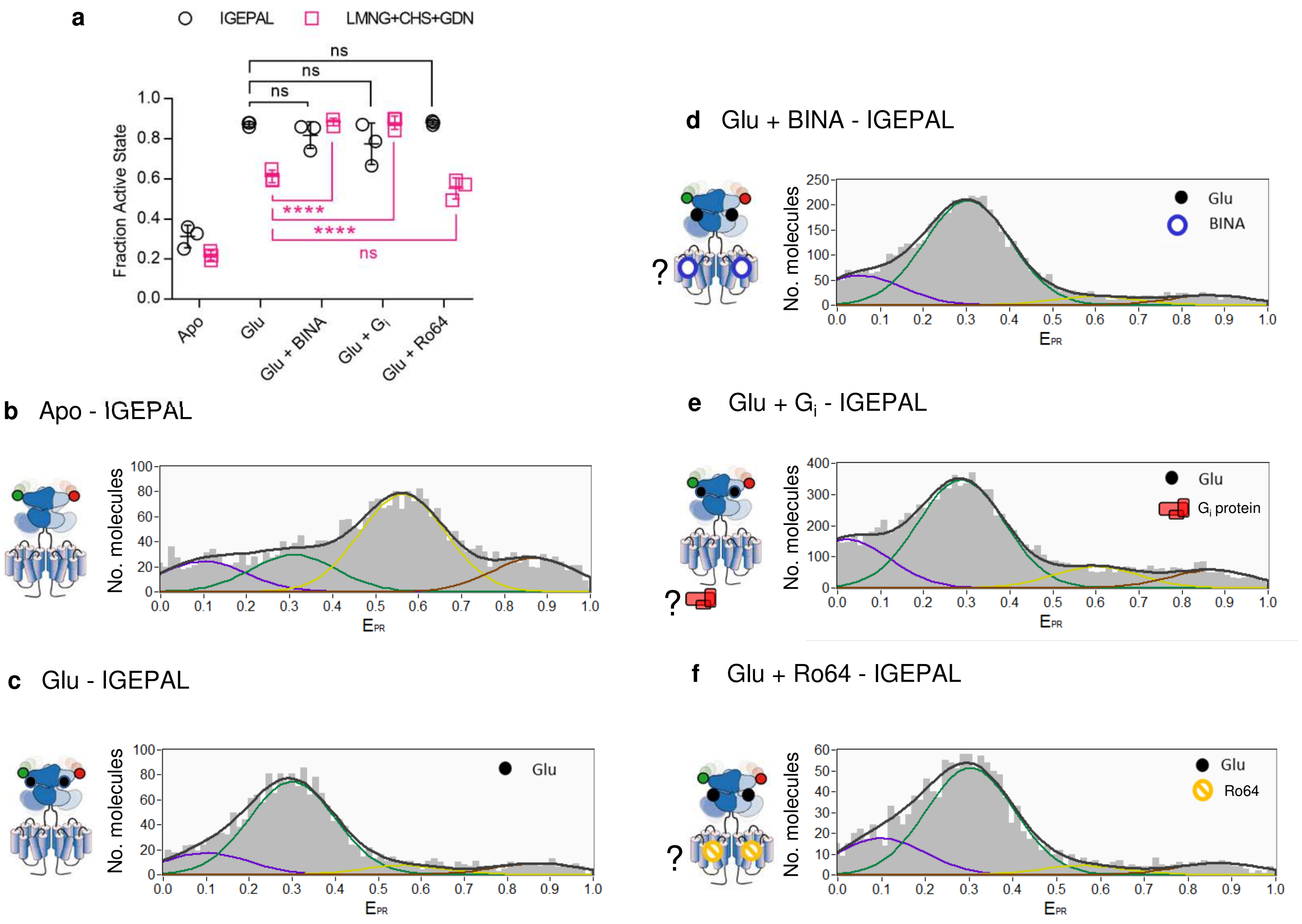

Supplementary Figure 24: Comparison of mGlu2 activation in IGEPAL and LMNG-CHS-GDN micelles. a) Comparison of the degree of activation in response to different ligands shows that in IGEPAL micelles Glu already leads to a maximal VFT reorientation, while BINA, G_i and Ro64 exhibit no effect. Data represent the mean of three biological replicates +/- SD. Statistical differences of pEC_{50} values for different conditions as indicated in LMNG-CHS-GDN (black) and IGEPAL (pink) compared to time Glu a one-way ANOVA with Sidak multiple comparisons test and are given as: LMNG-CHS-GDN $p_{Glu+BINA}$ = 0.76 (ns), p_{Glu+Gi} = 0.28 (ns), $p_{Glu+Ro64}$ = 0.9998 (ns); IGEPAL $p_{Glu+BINA}$ = 0.000008 (ns), p_{Glu+Gi} = 0.000009 (ns), $p_{Glu+Ro64}$ = 0.21 (ns). n = 3 independent biological samples examined over 3 independent experiments. b-f) Corresponding histograms of DA species, where contaminant species at long lifetimes are removed, fitted with fixed FWHM (0.2) and VHF (0.87) but variable VLF, LF and HF. As no effect of the histogram are observed, the binding of BINA, G_i and Ro64 to mGlu2 in IGEPAL cannot be confirmed.

MALPVTALLLPLALLLHAARPAAASGIDYKDDDDKAGIDAIMDKDCEMKRTTLDSPLGKLELSGCEQGLHEIKLL
GKGTSAADAVEVPAPAAVLGGPEPLMQATAWLNAYFHQPEAIEEFPVPALHHPVFQQESFTRQVLWKLLKVV
KFGEVISYQQLAALAGNPAATAAVKTALSGNPVPILIPCHRVVSSSGAVGGYEGGLAVKEWLLAHEGHRLGKP
GLGDIEGPAKKVLTLEGDLVLGGLFPVHQKGGPAEDCGPVNEHRGIQRLEAMLFALDRINRDPHLLPGVRLGA
HILDSCSKDTHALEQALDFVRASLSRGADGSRHICPDGSYATHGDAPTAITGVIGGSYSDVSIQVANLLRLFQIP
QISYASTSAKLSDKSRYDYFARTVPPDFFQAKAMAEILRFFNWTYVSTVASEGDYGETGIEAFELEARARNICV
ATSEKVGRAMSRAAFEGVVRALLQKPSARVAVLFTRSEDARELLAASQRLNASFTWVASDGWGALESVVAG
SEGAAEGAITIELASYPISDFASYFQSLDPWNNSRNPWFREFWEQRFRCSFRQRDCAAHSLRAVPFEQESKI
MFVVNAVYAMAHALHNMHRALCPNTTRLCDAMRPVNGRRLYKDFVLNVKFDAPFRPADTHNEVRFDRFGD
GIGRYNIFTYLRAGSGRYRYQKVGYWAEGLTLDTSLIPWASPSAGPLPASRCSEPCLQNEVKSVQPGEVCCW
LCIPCQPYEYRLDEFTCADCGLGYWPNASLTGCFELPQEYIRWGDAWAVGPVTIACLGALATLFVLGVFVRHN
ATPVVKASGRELCYILLGGVFLCYCMTFIFIAKPSTAVCTLRRLGLGTAFSVCYSALLTKTNRIARIFGGAREGA
QRPRFISPASQVAICLALISGQLLIVVAWLVVEAPGTGKETAPERREVVTLRCNHRDASMLGSLAYNVLLIALCT
LYAFKTRKCPENFNEAKFIGFTMYTTCIIWLAFLPIFYVTSSDYRVQTTTMCVSVSLSGSVVLGCLFAPKLHIILF
QPQKNVVSHRAPTSRFGSAAARASSSLGQGSGSQFVPTVCNGREVVDSTTSSL

Supplementary Figure 25: Amino acid sequence of human FLAG-SNAP-mGlu2 construct used in the present study. Red: Human CD8 signal peptide, green: FLAG-tag, pink: SNAP-tag, yellow: human metabotropic glutamate receptor 2.

a Glu + BINA - IGEPAL

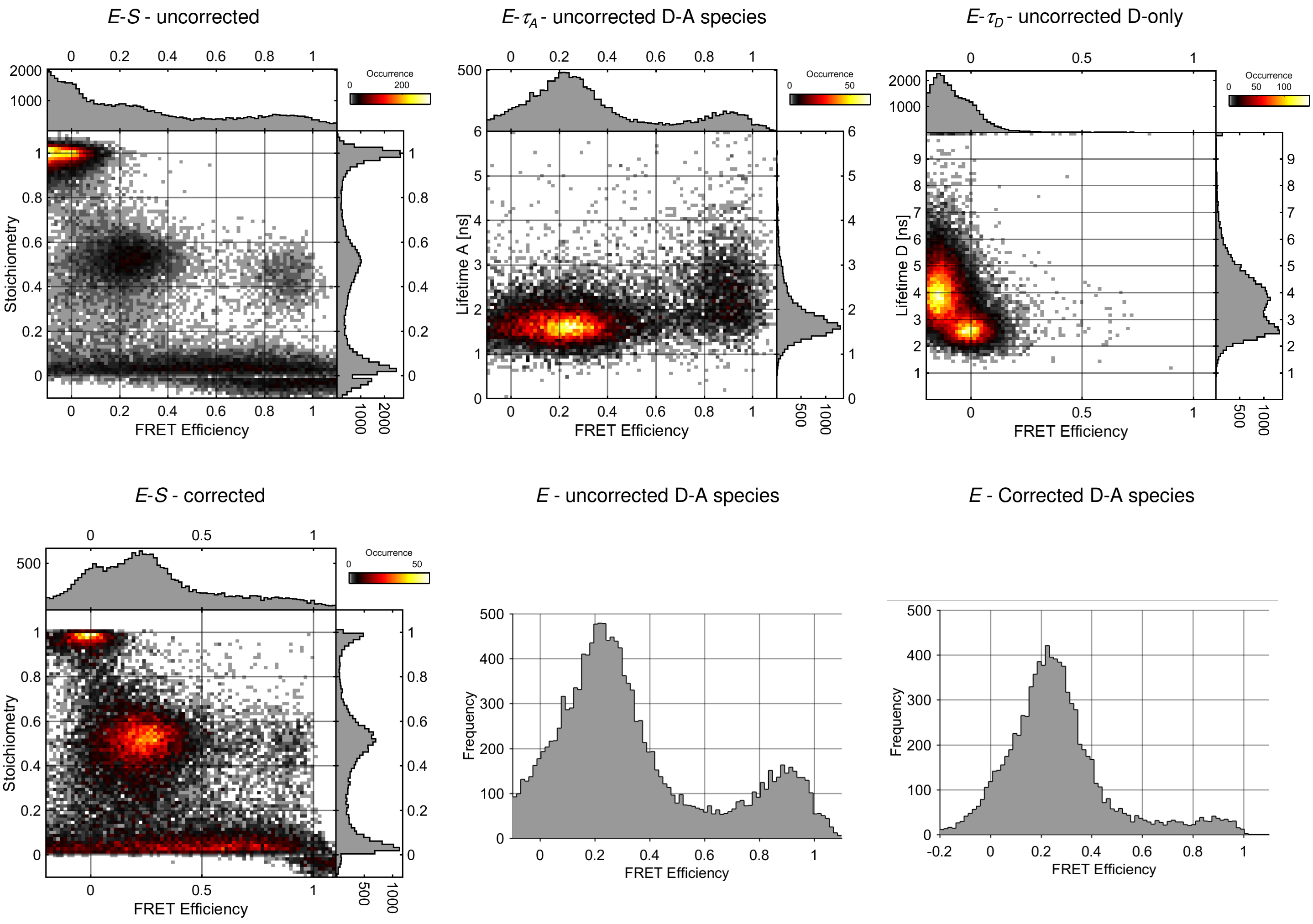

b Glu + BINA – LMNG+CHS+GDN

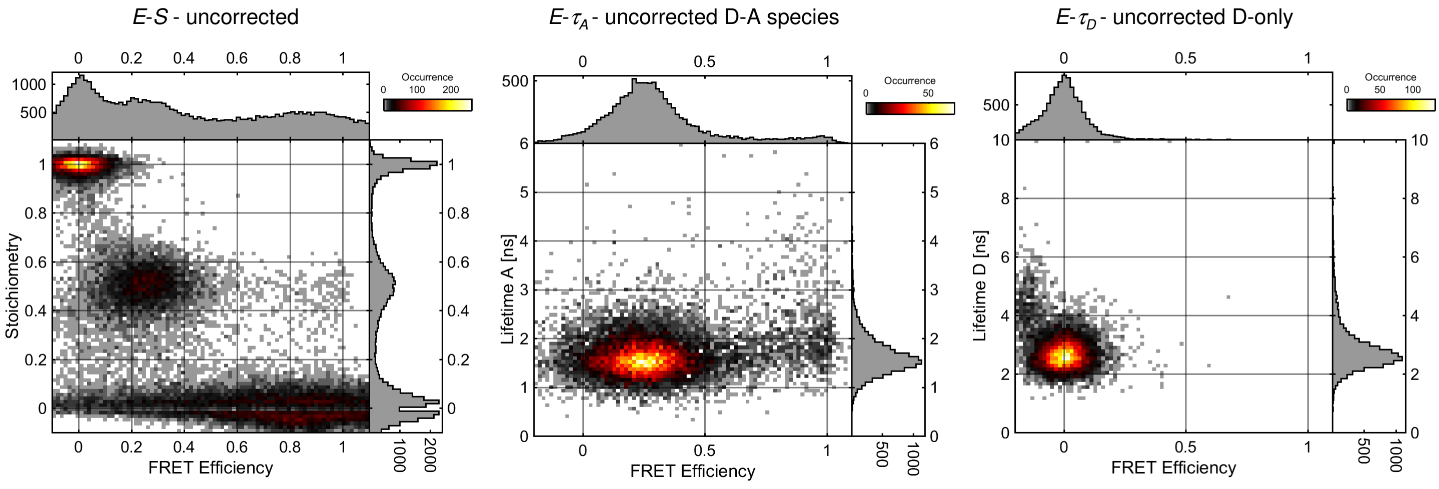

Supplementary Figure 26: Representative histograms showing fluorescent contaminations observed with IGEPAL (a) but not LMNG-CHS-GDN (b). In IGEPAL, a "high FRET" population (see E-S uncorrected) displaying a long lifetime value in the acceptor channel ($\tau_A > 2$ ns) that does not correspond to the d2 acceptor ($\tau_A = 1.8$ ns) is observed. This population is not present in LMNG-CHS-GDN (Compare E-τ_A - Uncorrected D-A species). Additionally, a donor-only-like population with a lifetime $\tau_d = 4$ ns longer than the used Cy3b donor (τ_D=2.8ns), and a spectral property that leads to less leakage in the acceptor channel than Cy3B (therefore the apparent FRET efficiency of this population is below zero (around -0.1)(see E-τ_D - Uncorrected D-only). This population is barely seen in LMNG-CHS-GDN. Removal of these two contaminants was only performed for the data with IGEPAL, by selecting molecules with τ_D<3ns and τ_A<2ns. This leads to corrected E-S histograms from which D-A species were selected and plotted in supplementary figure 24.
