# Supplementary material for: Allosteric modulators enhance agonist efficacy by increasing the residence time of a GPCR in the active state
Source: Nat Commun. 2021 Sep 14;12:5426. doi: 10.1038/s41467-021-25620-5 (PMC8440590; doi:10.1038/s41467-021-25620-5)
Supplement: Supplementary file 3 — Reporting Summary [file 41467_2021_25620_MOESM3_ESM.pdf]

nature portfolio

Corresponding author(s): Emmanuel Margeat and Jean-Philippe Pin

Last updated by author(s): Aug 20, 2021

Reporting Summary

Nature Portfolio wishes to improve the reproducibility of the work that we publish. This form provides structure for consistency and transparency in reporting. For further information on Nature Portfolio policies, see our Editorial Policies and the Editorial Policy Checklist.

Statistics

For all statistical analyses, confirm that the following items are present in the figure legend, table legend, main text, or Methods section.

n/a	Confirmed	
☐	☒	The exact sample size (*n*) for each experimental group/condition, given as a discrete number and unit of measurement
☐	☒	A statement on whether measurements were taken from distinct samples or whether the same sample was measured repeatedly
☐	☒	The statistical test(s) used AND whether they are one- or two-sided<br>*Only common tests should be described solely by name; describe more complex techniques in the Methods section.*
☒	☐	A description of all covariates tested
☒	☐	A description of any assumptions or corrections, such as tests of normality and adjustment for multiple comparisons
☐	☒	A full description of the statistical parameters including central tendency (e.g. means) or other basic estimates (e.g. regression coefficient) AND variation (e.g. standard deviation) or associated estimates of uncertainty (e.g. confidence intervals)
☐	☒	For null hypothesis testing, the test statistic (e.g. *F*, *t*, *r*) with confidence intervals, effect sizes, degrees of freedom and *P* value noted<br>*Give P values as exact values whenever suitable.*
☒	☐	For Bayesian analysis, information on the choice of priors and Markov chain Monte Carlo settings
☒	☐	For hierarchical and complex designs, identification of the appropriate level for tests and full reporting of outcomes
☒	☐	Estimates of effect sizes (e.g. Cohen's *d*, Pearson's *r*), indicating how they were calculated

Our web collection on statistics for biologists contains articles on many of the points above.

Software and code

Policy information about availability of computer code

Data collection	smFRET: spcm 9.85, B&H<br>LRET: PHERAstar V5.70 Edition 5, BMG Labtech
Data analysis	smFRET data analysis: Software package for multiparameter fluorescence spectroscopy, correlation and multiparameter fluorescence imaging, CAM Seidel, www.mpc.uni-duesseldorf.de/seidel/<br>PAM 1.3, D Lamb, www.cup.uni-muenchen.de/pc/lamb/software/pam<br>HTRF: MARS, BMG Labtech<br>mGlu2 structures: PYMOL 2.3.3<br>Graphs and histograms: PRISM 7.05, GraphPad

For manuscripts utilizing custom algorithms or software that are central to the research but not yet described in published literature, software must be made available to editors and reviewers. We strongly encourage code deposition in a community repository (e.g. GitHub). See the Nature Portfolio guidelines for submitting code & software for further information.

Data

Policy information about availability of data

All manuscripts must include a data availability statement. This statement should provide the following information, where applicable:

- Accession codes, unique identifiers, or web links for publicly available datasets
- A description of any restrictions on data availability
- For clinical datasets or third party data, please ensure that the statement adheres to our policy

Source data are provided with this paper. Raw data of smFRET acquisitions will be provided upon reasonable request.

Field-specific reporting

Please select the one below that is the best fit for your research. If you are not sure, read the appropriate sections before making your selection.

☒ Life sciences ☐ Behavioural & social sciences ☐ Ecological, evolutionary & environmental sciences

For a reference copy of the document with all sections, see nature.com/documents/nr-reporting-summary-flat.pdf

Life sciences study design

All studies must disclose on these points even when the disclosure is negative.

Sample size	All presented data where derived from at least 3 independent biological samples
Data exclusions	no data were excluded unless obvious experimental errors where encountered
Replication	LRET: measurements were repeated on 3-4 independent biological replicates,where each condition was measured in 3 technical replicates<br>smFRET: measurements were repeated on 3 independent biological replicates
Randomization	Randomization was not applicable as samples were homogenous - smFRET measurements where performed on freshly solubilized receptors, stored on ice for up to 48h, different ligand conditions were measured at different intervals of storaged post-solubilization for different biological replicates
Blinding	Blinding was not applicable as specific protein samples were prepared, checked for quality and analyzed under defined conditions.

Reporting for specific materials, systems and methods

We require information from authors about some types of materials, experimental systems and methods used in many studies. Here, indicate whether each material, system or method listed is relevant to your study. If you are not sure if a list item applies to your research, read the appropriate section before selecting a response.

Materials & experimental systems

n/a	Involved in the study	
☒	☐	Antibodies
☐	☒	Eukaryotic cell lines
☒	☐	Palaeontology and archaeology
☒	☐	Animals and other organisms
☒	☐	Human research participants
☒	☐	Clinical data
☒	☐	Dual use research of concern

Methods

n/a	Involved in the study	
☒	☐	ChIP-seq
☒	☐	Flow cytometry
☒	☐	MRI-based neuroimaging

Eukaryotic cell lines

Policy information about cell lines

Cell line source(s)	HEK293T cells (ATCC CRL-3216, LGC Standards S.a.r.l., France), Sf9 insect cells (Life Technologies)
Authentication	Cell lines were not authenticated.
Mycoplasma contamination	Cell lines were tested negative for mycoplasma.
Commonly misidentified lines (See ICLAC register)	No commonly misidentified cell lines were used.
